# Overlooked Mountain Rock Pools in Deserts Are Critical Local Hotspots of Biodiversity

**DOI:** 10.1371/journal.pone.0118367

**Published:** 2015-02-25

**Authors:** Cândida Gomes Vale, Stuart L. Pimm, José Carlos Brito

**Affiliations:** 1 CIBIO/InBIO, Centro de Investigação em Biodiversidade e Recursos Genéticos da Universidade do Porto, Instituto de Ciências Agrárias de Vairão, R. Padre Armando Quintas, Vairão, Portugal; 2 Departamento de Biologia da Faculdade de Ciências da Universidade do Porto, Rua Campo Alegre, Porto, Portugal; 3 Nicholas School of the Environment, Duke University, Durham, North Carolina, United States of America; Institute of Agronomy, University of Lisbon, PORTUGAL

## Abstract

**Background:**

The world is undergoing exceptional biodiversity loss. Most conservation efforts target biodiversity hotspots at large scales. Such approach overlooks small-sized local hotspots, which may be rich in endemic and highly threatened species. We explore the importance of mountain rock pools (gueltas) as local biodiversity hotspots in the Sahara-Sahel. Specifically, we considered how many vertebrates (total and endemics) use gueltas, what factors predict species richness, and which gueltas are of most priority for conservation. We expected to provide management recommendations, improve local biodiversity conservation, and simultaneously contribute with a framework for future enhancement of local communities’ economy. The identification of local hotspots of biodiversity is important for revaluating global conservation priorities.

**Methodology/Principal Findings:**

We quantified the number of vertebrate species from each taxonomic group and endemics present in 69 gueltas in Mauritania, then compared these with species present in a surrounding area and recorded in the country. We evaluated the predictors of species number’s present in each guelta through a multiple regression model. We ranked gueltas by their priority for conservation taking into account the percentage of endemics and threats to each guelta. Within a mere aggregate extent of 43 ha, gueltas hold about 32% and 78% of the total taxa analysed and endemics of Mauritania, respectively. The number of species present in each guelta increased with the primary productivity and area of gueltas and occurrence of permanent water. Droughts and human activities threaten gueltas, while 64% of them are currently unprotected.

**Conclusion/Significance:**

Gueltas are crucial for local biodiversity conservation and human activities. They require urgent management plans in Mauritania’s mountains. They could provide refugia under climate change being important for long-term conservation of Sahara-Sahel biodiversity. Given their disproportional importance in relation to their size, they are local hotspots of biodiversity deserving global attention.

## Introduction

The world is undergoing exceptional biodiversity loss [1]. Most conservation efforts target biodiversity hotspots because they constitute areas of exceptional endemic richness that are undergoing significant habitat loss [2–4]. Identification of hotspots of richness and general understanding of richness-environment relationships is of major importance. These evaluations are mostly global or continental [2], while the local patterns of species richness, endemism and rarity are less well understood [5]. Large-scale assessments are likely to miss regional patterns and small-sized areas with large number of endemics that could constitute local hotspots [6–8]. Given that most land-use transformation and management decisions are made at local or regional scales, overlooking local hotspots may constitute a serious deficiency in biodiversity conservation.

The common perception of deserts and arid regions is that they constitute remote areas of low diversity when compared to other biomes. No desert is listed in the global biodiversity hotspots [9]. In fact, the world’s largest warm desert, the Sahara, together with the neighbouring arid Sahel, have patchily distributed species and a relatively high number of endemics. These species are often restricted to small and fragile humid habitats [10]. Surrounded by sandy areas, isolated and residual water features (oases, lakes and seasonal rivers) act as refugia for relict populations and constitute places where unique species evolve [10–12]. Indeed, water availability strongly predicts communities’ distribution and species richness in drylands [13–14]. Desertification and human activities affect water availability and threaten these water features [10]. As such, those within the Sahara-Sahel may constitute local hotspots of biodiversity under threat. Despite the conservation importance of water features in the Sahara-Sahel, we know little about their species richness, particularly their endemics, and the threats affecting them.

In Mauritania, endemic species and range-margins populations of different biogeographic origin are restricted to mountain rock pools, locally known as gueltas [15–17]. The country is located in biogeographic crossroad between Palaearctic and Afro-tropical ecoregions [18] and mountains disrupt the latitudinal gradient in climate and habitat of the region [19]. Gueltas are small (from 0.01 to 5ha) and water availability is mostly seasonal. In many gueltas, water is only available during the rainy season (July to September), when torrential waterfalls fill up the pools [16]. This makes them susceptible to different threats and vulnerable to future climate change. The droughts of the 1970s [19–20] have caused some gueltas of northernmost Mauritania dry out [15] and to decrease the nomadic lifestyle in favour of sedentary habits around permanent water bodies. Presently, herdsmen overexploit those gueltas, producing water-shortage during the dry season, faecal contamination by domestic animals, and increased activities for excavating pools or pumping water [16, 21]. Improved knowledge on species richness of gueltas and types of threats affecting them is important to establish priorities for their conservation.

Here, we explore the importance of gueltas as local biodiversity hotspots. We addressed three specific questions. First, how many species use gueltas? We considered how many vertebrates (total and endemics) use gueltas and compared these with those present in the mountains of Mauritania and in the country. We expected that a large proportion of vertebrates, particularly endemics, would live in gueltas. Secondly, what are the predictors of species richness? We expected that species richness would correlate with water availability. Thirdly, which gueltas are the highest priority for conservation? We identified and quantified threats to each guelta and rank gueltas according to their conservation priority. Revaluation of conservation priorities that include society needs in countries covered by deserts and arid regions are a global challenge [22]. As such, knowledge about biodiversity and threats in gueltas of Mauritania is important for local sustainable resource use that policy makers might use as framework for future enhancement of local communities’ economy. The identification of local hotspots of biodiversity is important to revaluate global priorities, being a valuable contribution for global biodiversity conservation.

## Materials and Methods

### Ethics statement

Fieldwork was developed with permission from the Ministére Délégué auprès du Premier Ministre Chargé de l’Environnement, Nouakchott (Permit: 460/MDE/PNBA). This permit was valid for the entire country and no specific permissions were required for any specific locality. Analyses were done at a CITES registered laboratory: 13PT0065/S. Field collection and handling practices were approved by the Committee of Animal Experimentation of the University of Porto (Portugal) under the Directive 2010/63/EU of the European Parliament. No animal was sacrificed and there were no animal husbandry, experimentation and care/ welfare concerns.

### Study area

**Fig 1:**
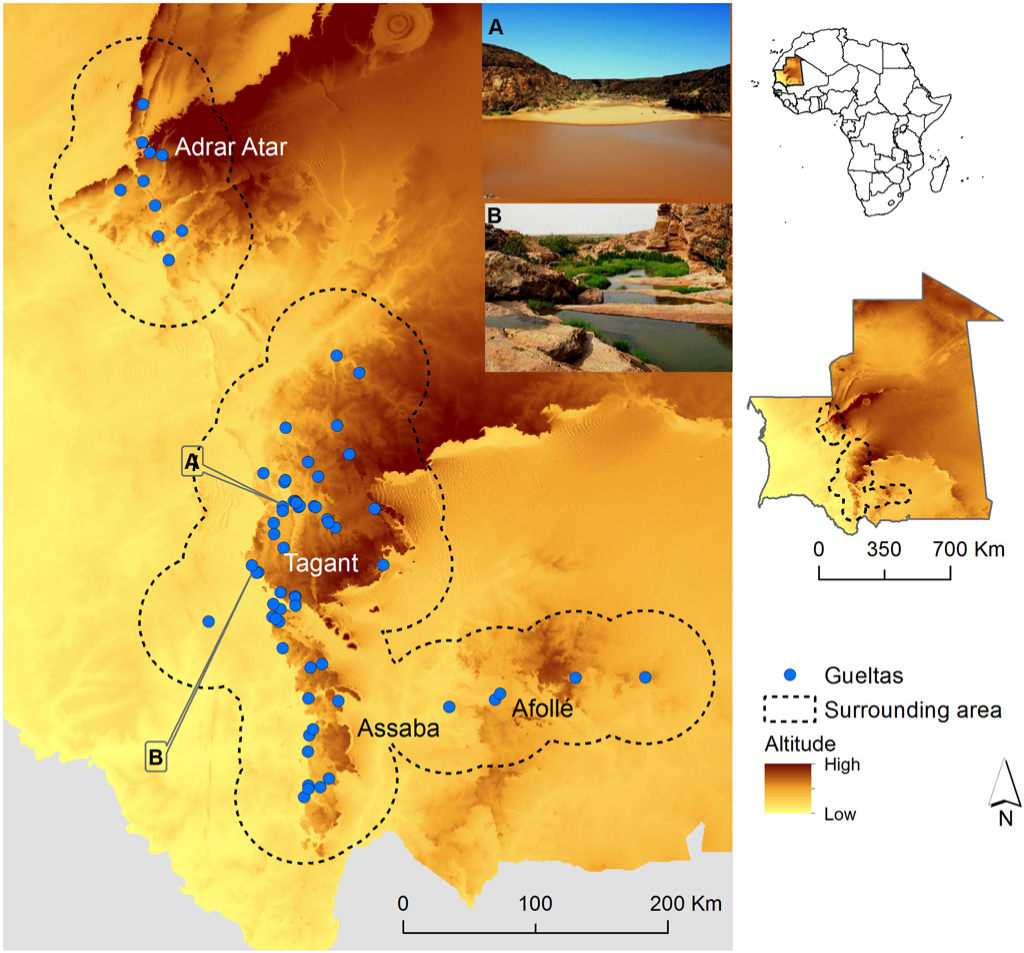
Study area and the locations of the gueltas. Example of two gueltas: A) Guelta Tartêga, and B) Guelta Garaouel. Black dashed line limits the surrounding area. Insets display location of Mauritania within the African context (top) and location of the surrounding area within Mauritania (bottom).

The study area is in West Africa between 15.8°N and 20.6°N and west of 9.5°W, and comprises the Mauritanian mountains of Adrar Atar, Tagant, Assaba and Afollé (Fig. 1). The Adrar Atar is the northernmost, with vegetation of Palearctic affinity; the harsh Sahara desert surrounds it. The southern Tagant, Assaba and Afollé mountains have wetter climate and vegetation of Sudanese affinity. Gueltas are mostly upstream of narrow valleys at the base of mountains (see S1 Appendix). The total area occupy by gueltas in Mauritania is approximately 43 ha (0.00004% of the total area of Mauritania and 0.0007% of its mountains).

### Fieldwork and species observations

Starting in 2007, we have completed seven overland field expeditions to Mauritania, during which we visited 69 gueltas (see S1 Table and S1 Appendix). We recorded the location and area of each guelta with a GPS (WGS84 datum). Field missions ran annually from September to December (after the rainy season), except in 2009 (March-May; dry season peak). Each guelta was sampled by at least 3 persons using several distinct methodologies: visual inspection, deep-netting, Sherman traps, camera traps, indirect observations (faeces, footprints, tracks or burrows) and night sampling of the water and margins with lamps. The effort was about 237 man-hours in total and 3.43 man-hours in each guelta. We collected 4200 geo-referenced observations from 107 vertebrates, including fishes, amphibians, reptiles and mammals. For comparison, we compiled taxonomic reference lists of vertebrates in Mauritania for fishes [15, 23–26], amphibians [27], reptiles [28], and mammals [29].

We quantified, by direct observation in the field, the type and number of threats affecting each guelta, following the nomenclature used by IUCN guidelines for listing threats [30].

### Quantification of species richness

To quantify species richness in gueltas, we first designed buffers around each guelta, using the “Buffers” tool of ArcGIS 10.0.0. Buffer size was set according to potential dispersal abilities of each taxonomic group: 2 km for fishes, amphibians and reptiles and 5 km for mammals. To quantify the number of species that occur in mountains and that could potentially reach gueltas, we defined an area that bounded gueltas with a buffer of 50km (likely corresponding to the maximum dispersal distance of any species occurring in gueltas) and call it the "surrounding area" (Fig. 1). We quantified the number of species from each taxonomic group and endemics present in: 1) the gueltas; 2) in the surrounding area; and 3) in Mauritania. We then compared number of species observed in each guelta with the number of species quantified in the surrounding area, and recorded in Mauritania. We considered as Mauritanian endemics, those species with at least 75% of the global range located inside Mauritania. This includes two described species and seven taxa delimited based on genetic evidences pointing towards a reciprocally monophyletic status and long divergence time (authors’ unpublished data) [31]. We used Chi-Square tests to identify significant differences in the number of species among mountains from each taxonomic group and from each IUCN conservation category.

### Predictors and analyses

We selected environmental factors to evaluate their relationships with observed species richness in each guelta. They included: 1) the area of the guelta (m^2^) estimated in the field with a GPS (see above) 2) the Normalized Difference Water Indexes to detect permanent and seasonal water (NDWI_P and NDWI_S), respectively, at 1 arc second resolution [32]; and 3) a Normalized Difference Vegetation Index (NDVI) time-series from the period between 2003 and 2011, at 30 arc second resolution [33]. For all indexes, we calculated several measures of ecological significance: 1) annual maximum; 2) annual mean; 3) annual standard deviation; and 4) the maximum annual average. We initially explored the effects of latitude, but then excluded this variable because it so highly correlates with NDVI values (r_s_ > 0.80, p = 0.00).

We evaluated all of the predictors (including all indexes measures) of the number of species present in each guelta through a multiple regression model (GLZ), using the MuMIn package in R software v. 3.0.2. Models were ranked according to their Akaike’s information criterion value. Each model’s support was estimated through the difference in AICc with respect to the top-ranked model (ΔAICc). AICc rather than AIC is appropriate when there are too many parameters relative to sample size [34–35]. We used the best model to determine the importance of predictors and their significance for each model.

### Quantification of threat and conservation priorities

We ranked gueltas by their priority for conservation taking into account the percentage of endemic species and threats to each guelta. We plotted the percentage of endemics and threats and reclassified gueltas according their priority for conservation. We defined three levels of priority: 1) low—gueltas with low percentage of endemics even if they were vulnerable to high levels of threats; 2) important- gueltas with high percentage of endemics but less vulnerable to different threats; and 3) priority—gueltas with high percentage of endemics and threats. Finally, we performed Chi-Square tests to test for differences in the number of threats among gueltas of each mountain.

To quantify the number of gueltas currently protected (total and by priority levels), we intersected the location of gueltas with the protected areas of Mauritania.

## Results

### Numbers of species

Some 59 vertebrate species use gueltas. This represents a significant portion of all vertebrates of Mauritania and of the surrounding area (Table 1 and see S2 and S3 Tables). There are no significant differences in the number of species using gueltas among mountains (p = 0.93). The number of species observed in each guelta did not increase with sampling effort (r_s_ = 0.18 p = 0.14).

**Fig 2:**
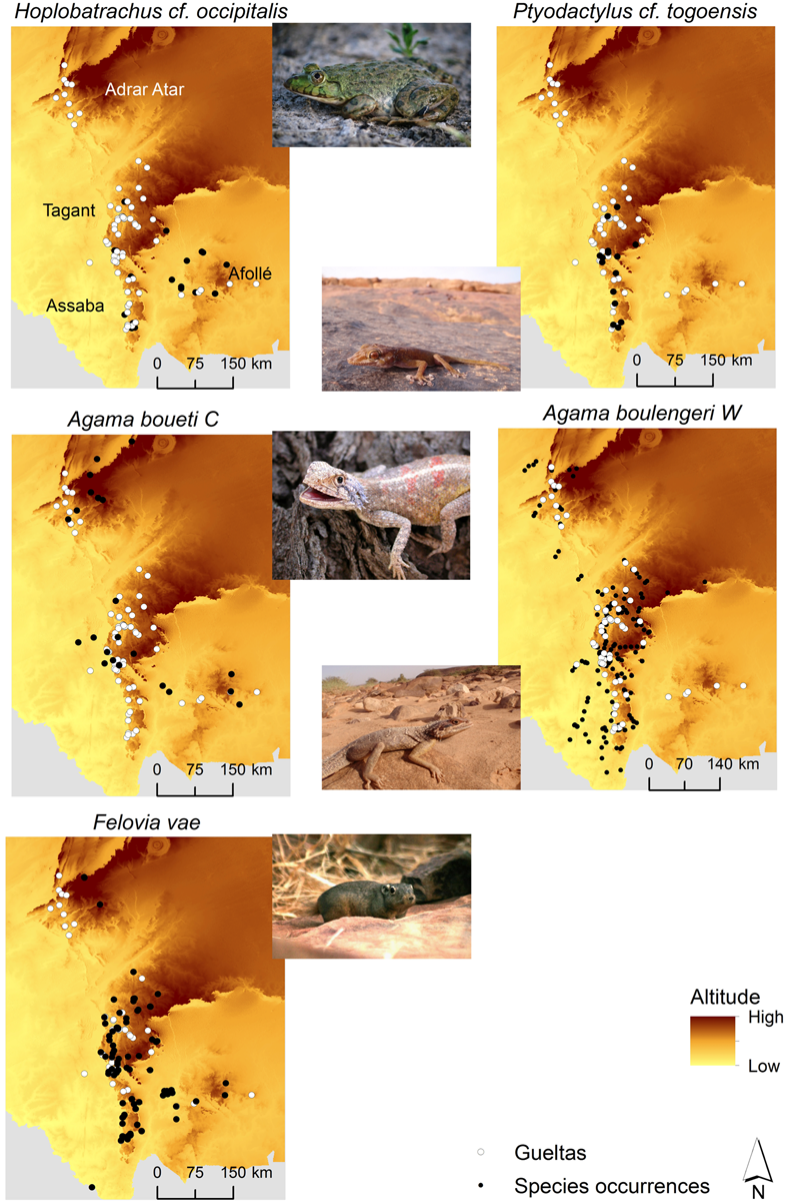
Known distribution of endemic taxa of Mauritania observed in gueltas.

Gueltas held 78% of the Mauritanian endemics (Table 1). Gueltas of Adrar Atar exhibited fewer endemic species than gueltas of the southern mountains (Table 1). There are endemic species that are present in all mountains, such as *Felovia vae* (Fig. 2). Other endemics were restricted to the southern gueltas (*Ptyodactylus cf*. *togoensis* and *Hoplobatrachus cf*. *occipitalis*) (Fig. 2). The endemic *Pristurus adrarensis* is restricted to Adrar Atar, but it was not observed in gueltas (S2 Table).

**Table 1:** Sum of taxa (Σ) quantified in gueltas by taxonomic group and IUCN status.

	Σ Adrar	Σ Tagant	Σ Assaba	Σ Afollé	Σ Gueltas	Σ SA (%G)	Σ Mau (%G)
Fishes	2	2	5	3	5	7 (71)	18 (28)
Amphibians	2	3	6	3	7	7 (100)	11 (64)
Reptiles	5	16	17	10	24	41 (56)	79 (30)
Mammals	3	13	14	13	23	29 (79)	78 (29)
Total	12	34	42	29	59	86 (69)	186 (32)
							
Mau Endemic	2	5	6	4	7	9 (78)	9 (78)
							
DD	1	2	2	2	2	3 (67)	7 (29)
NE	5	13	18	11	23	40 (58)	69 (33)
LC	6	18	21	15	33	40 (83)	96 (34)
NT	0	1	1	1	1	1 (100)	6 (17)
VU	0	0	0	0	0	2 (0)	7 (0)
CR	0	0	0	0	0	0 (0)	1 (0)

Sum of taxa and endemic taxa (Mau Endemic) quantified in the surrounding area (SA) and in Mauritania (Mau), and percentage of those present in gueltas (%G). DD: Data deficient; NE: Not evaluated; LC: Least concern; NT: Near threatened; VU: Vulnerable; CR: Critically Endangered.

We did not observe any species in gueltas that IUCN deems to be threatened (Table 1). A few species IUCN deems Data Deficient and Near Threatened species from Mauritania used gueltas, but the number increased when we compared with the species of the surrounding area (100%). A large proportion of species using gueltas remain Not Evaluated by IUCN (Table 1). There were no significant differences between mountains in the number of species from each IUCN conservation category (p = 1).

### Predictors of the numbers of species, quantification of threats and conservation priorities

We selected the best model to describe relationships between species richness and predictors according to the lower AICc (Table 2). The number of species present in each guelta increased with both productivity (maximum of the annual average NDVI), occurrence of permanent water (annual average NDWI_P), and area of the guelta, and declined with the occurrence of seasonal water (maximum NDWI_S; Table 2). Droughts and temperature extremes threatened all gueltas (100%, Table 3) and extraction of water for domestic use and nomadic grazing were also frequent (81% and 80%, respectively). There were no significant differences between mountains in the number of threats observed in each guelta (p = 0.504). Human related threats increase with the increasing area of gueltas (r_s_ = 0.3, p = 0.01).

**Table 2:** Measures of the predictors most related with the species richness in gueltas (GLZ).

	β	Std. Error	z value	Pr(>|z|)	AICc	ΔAIC	Wi
AREA	0.00	0.00	2.76	0.01**			
NDVImax_avg	0.00	0.00	6.15	0.00***			
NDWI_Pavg	6.70	1.60	4.19	0.00***	379.87	0	0.23
NDWI_Psd	4.85	1.97	2.47	0.01*			
NDWI_Smax	-3.16	0.69	-4.57	0.00***			

Significance codes: p < 0.0001 ***;

p < 0.001 **;

p < 0.01 *.

NDVImax_avg: Maximum of the annual average of Normalized Difference Vegetation Index; NDWI_Pavg and NDWI_Psd: Annual average and standard deviation of the annual average of Normalized Difference Water Indexes of permanent water, respectively; and NDWI_Smax: Maximum of Normalized Difference Water Index of seasonal water.

**Table 3:** Number and percentage of gueltas affected by each threat.

Threats		Σ Gueltas (%)
2.3.1	Nomadic grazing	55 (80)
2.3.2	Small-holder grazing, ranching or farming	24 (35)
5.1.1	Intentional use (species being assessed is the target)	10 (14)
5.4.1	Intentional use: subsistence/small scale (species being assessed is the target)	24 (35)
7.2.1	Abstraction of surface water (domestic use)	56 (81)
7.2.3	Abstraction of surface water (agricultural use)	21 (30)
9.3.4	Pollution: Type Unknown/Unrecorded	51 (74)
9.4	Pollution: Garbage & solid waste	23 (33)
10.3	Avalanches/landslides	26 (38)
11.2	Droughts	69 (100)
11.3	Temperature extremes	69 (100)

Codes follow the IUCN Threats Classification Scheme [30].

**Fig 3:**
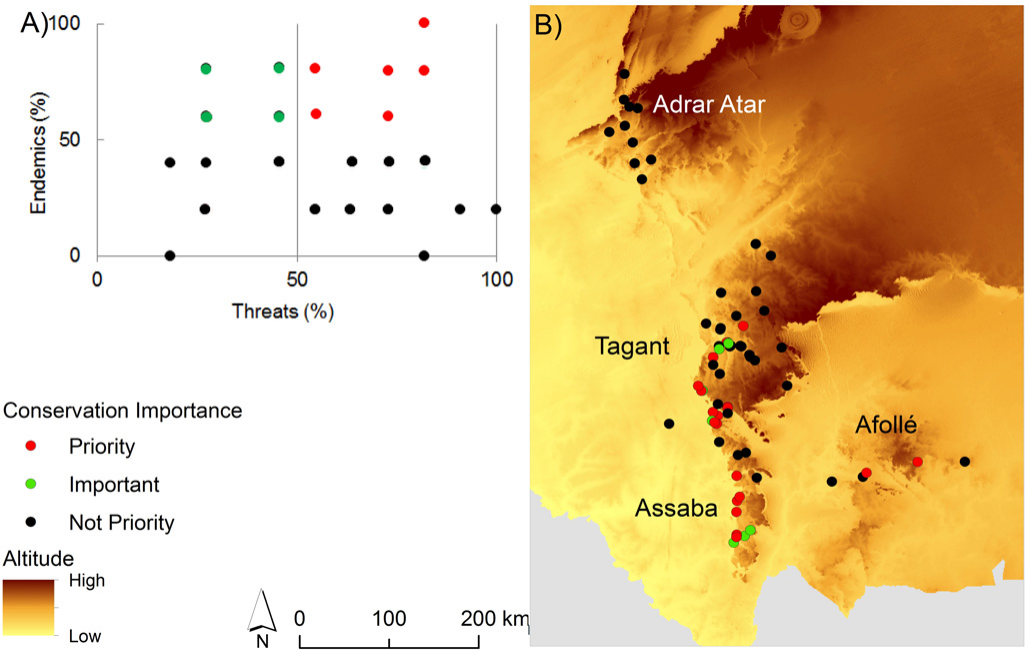
Priority gueltas for conservation. A) Ranking of conservation importance of gueltas taking into account the percentage of endemics and threats. Red dots represent priority gueltas for conservation (many endemics and threats); green dots represent important gueltas for conservation (many endemics and few threats) and black dots represent less important gueltas for conservation (few endemics). B) Location of all gueltas coloured by the importance for conservation.

There were significant differences in priorities for conservation between gueltas in each mountain (p = 0.009). The most priority gueltas for conservation were located in the southern mountains: Tagant, Assaba and Afollé (Fig. 3). From the 69 gueltas, only 25 are currently included in one Ramsar site in the Tagant plateau while remaining gueltas (~64%) do not have any legal protection status. From the 25 gueltas protected by the Ramsar site, 16% and 12% were ranked as priority and important for conservation, respectively. About 80% of the gueltas (N = 16) identified as priorities are unprotected.

## Discussion

Gueltas are special places and are disproportionately important for their tiny size. The 69 gueltas contained 32% of the analysed vertebrates of Mauritania and 78% of the country’s endemics in an area representing only 0.00004% of the country. As such, gueltas are local hotspots of biodiversity deserving global attention. IUCN deems none of the species as threatened, but a large proportion has not yet been evaluated. The observed lack of threatened species may reflect knowledge gaps about desert biodiversity [10], suggesting that we need more complete evaluations of conservation status.

The number of endemics is not similar in each mountain. The gueltas of southern mountains house more endemic species in comparison to the gueltas of Adrar Atar. For instance, the amphibian *Hoplobatrachus cf*. *occipitalis* is apparently restricted to the southern mountains. Lower diversity of endemics in Adrar Atar may be related to latitudinal gradients in climate and habitat, environmental tolerances of each species, and past Sahara-Sahel climatic oscillations [10]. These oscillations induced a series of extinctions and recolonisations and perhaps adaptation events that have shaped species composition in each mountain. Several species from Afro-tropical region have expanded throughout the Sahara-Sahel during wetter periods and then remained in mountain refugia during dry periods [10]. Currently, gueltas are refugia for several species due to the region’s aridity. Yet, these aquatic systems, as evolutionary and ecological refuges in arid environments are likely to constitute future refuges under global and regional climatic changes [36]. As future climate models predict more frequent or severe droughts for the region [20, 37], it is likely that gueltas will also constitute refugia under future climate change, particularly for water-dependent species.

High primary productivity, presence of permanent water, and area of gueltas are the best predictors of the numbers of species. Given the harsh surrounding environment, gueltas with high primary productivity likely held more species, as the amount of energy available is a major determinant of species richness [38]. Studies made in permanent and ephemeral streams subjected have found strong correlations between specie’s numbers and maximum NDVI [38]. Larger gueltas likely create opportunities and habitat conditions for more species. Permanent water is important for fishes, crocodiles and mammals in the gueltas of Mauritania [10, 15, 17] as well as for relict populations of Afro-tropical fishes in the gueltas of the Tibesti mountain of Chad [12]. In fact, gueltas could be the only source of water over large distances across the Sahara-Sahel. Permanent water features play a vital role in the conservation of local biodiversity, particularly in arid environments worldwide [36]. Research efforts should quantify species richness and threats in gueltas in other Sahara-Sahel mountains [10].

Droughts and temperature extremes affect all gueltas. Human disturbance is important and larger gueltas are more vulnerable to human pressures. Local communities base their economy on the exploration of the water and surrounding habitats of gueltas. Rock engravings provide clear historical evidences of their human use since the Neolithic [39]. Moreover, human activities in gueltas likely increased after the droughts of 1970’s. As an example, the Tagant plateau currently houses a population of agriculturalists and herdsmen, leading to activities of excavating pools and pumping water and to faecal contamination of water [21, 39]. The importance of gueltas for both biodiversity conservation and human activities suggest that the conservation of these local hotspots should incorporate the management of water as a resource.

The southern mountains (Tagant, Assaba and Afollé) hold the gueltas with the highest concentrations of endemics and, at the same time, the most threatened gueltas. The importance of the Tagant plateau has been recognized and the “Lac Gabou et le Réseau Hydrographique du Plateau du Tagant” have been classified as a Ramsar site [39]. The site only covers 20% of the top-priority gueltas for conservation, however. In fact, 64% of the total gueltas are unprotected and the current protected area network of the country fails to adequately preserve gueltas and its biodiversity. Designation of more protected areas should be considered for the gueltas this study identifies as most important.

Gueltas are special places for the conservation of biodiversity and simultaneously crucial for local communities activities. Mauritania is listed by FAO as of Low-Income Food-Deficit Country [40] and its Gross national income (GNI) per capita was 2.118 $US in 2010 (for instance: USA was 47.094 $US [41]). Livestock play an important role in the country, contributing around 10–15% of the GNI of the country [42]. As such, the allocation of land to biodiversity conservation competes with other land uses and societal needs. We believe that the best strategy to protect gueltas is to rank priorities for conservation and design a reserve network that would enhance both the protection of biodiversity and a sustainable development. Mauritania has been listed among the top countries with highest return-on-investment [43]. A conservation plan should reveal the economic benefits and rewards that local communities can derive from ecosystem services, such as sustainable resource use, ecotourism, and public health. For instance, conduction channels could feed troughs distant from the guelta, thus reducing current human and livestock pressure. Such infrastructures would also allow decreasing faecal contamination of the water, contributing to public health. Pollution threatens water quality in Mauritania [44] and diarrhoea is prevalent in the south of the country [45]. Organised ecotourism is possible, as the most accessible gueltas have crocodiles (particularly at Matmâta, see S1 Appendix) and groups of travellers regularly visit the ruins of Ksar el Barka [39]. To meet these proposals, funds might be obtained from the Global Environment Facility of the World Bank. Combining conservation priorities that factor in both biodiversity value and conservation management investments provides a new lens for setting global conservation priorities [43]. As such, a conservation programme should be implemented to protected these local hotspots and therefore, improve global biodiversity conservation.

Our study demonstrates the importance of gueltas as local biodiversity hotspots and it lays the foundations to build an effective conservation plan to protect them. Due to the current lack of information, complementary studies are still needed. The taxonomy and systematics of many reptiles and fishes is still uncertain, and molecular tools should be applied to identify conservation units. Often allied with water pools in deserts, aquatic macro invertebrates and bats also need further investigation. As aquatic macro invertebrates are considered good indicators of water quality in arid environments [46], their identification could also contribute to manage potential disease-vector species in gueltas. Physicochemical parameters (i.e. temperature, pH, conductivity, dissolved oxygen and turbidity); nitrates and nitrites concentrations, organic suspend matter and concentrations of chlorophyll a (a measure of algal biomass) should also be conducted to quantify water quality parameters related to public health. Studies about aquatic flora are also required.

## Conclusion

Gueltas are tiny places that hold high number of species, including endemics, and they are vulnerable to droughts and human activities. Given their disproportional importance for their size, they constitute local hotspots of biodiversity, overlooked by global assessments. Moreover, they could provide refugia under climate change, so they are crucial for long-term conservation of Sahara-Sahel biodiversity. Reserve networks that enhance both biodiversity conservation and human activities should be implemented in Mauritanian mountains. The observed value of gueltas of Mauritania as local hotspots are well representative of all gueltas of the Sahara-Sahel mountains as well as other small yet rich places surrounded by hostile habitats.

## Supporting Information

S1 Appendix Photographs of gueltas (PDF)

S1 Table Description of Gueltas (PDF)

S2 Table Taxa present in each guelta. (PDF)

S3 Table Sum of species and endemics present in each guelta by taxonomic group and IUCN status. (PDF)
