# Supplementary material for: Overlooked Mountain Rock Pools in Deserts Are Critical Local Hotspots of Biodiversity
Source: PLoS One. 2015 Feb 25;10(2):e0118367. doi: 10.1371/journal.pone.0118367 (PMC4340953; doi:10.1371/journal.pone.0118367)
Supplement: S1 Appendix — (PDF) [file pone.0118367.s001.pdf]

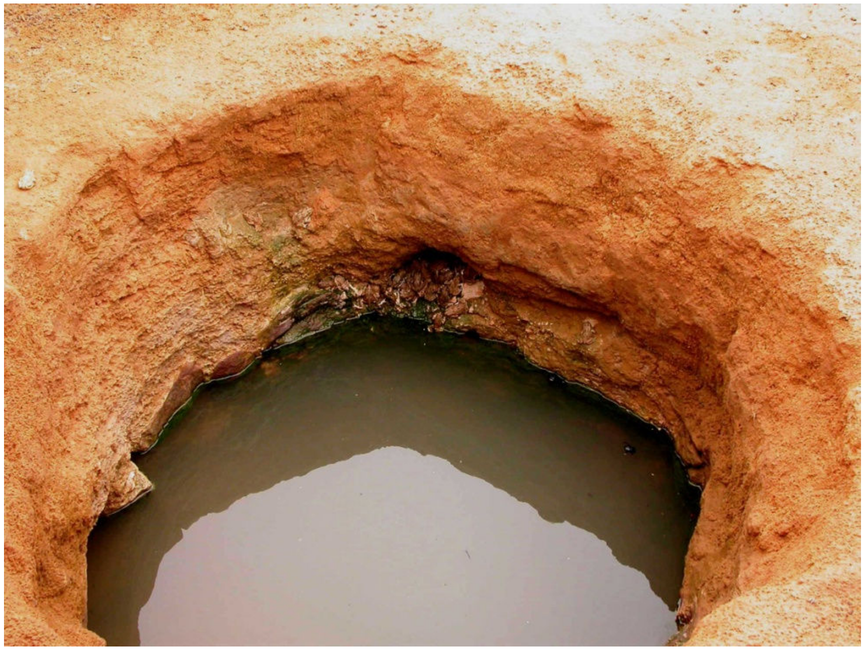

G01 Agmeimîne
Guelta (not observable) was dry at time of visit. *Amietophrynus xeros* were observed inside a well beside the *guelta.*
Photo: F Martínez-Freiría

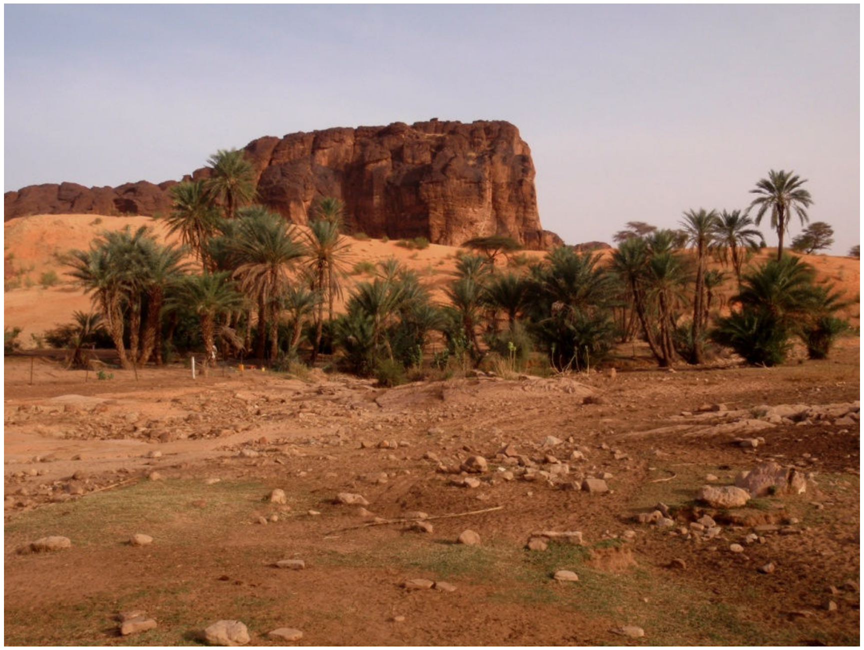

G02 Ain El Berbera
Guelta (not observable) is surrounded by palm trees
Photo: JC Brito

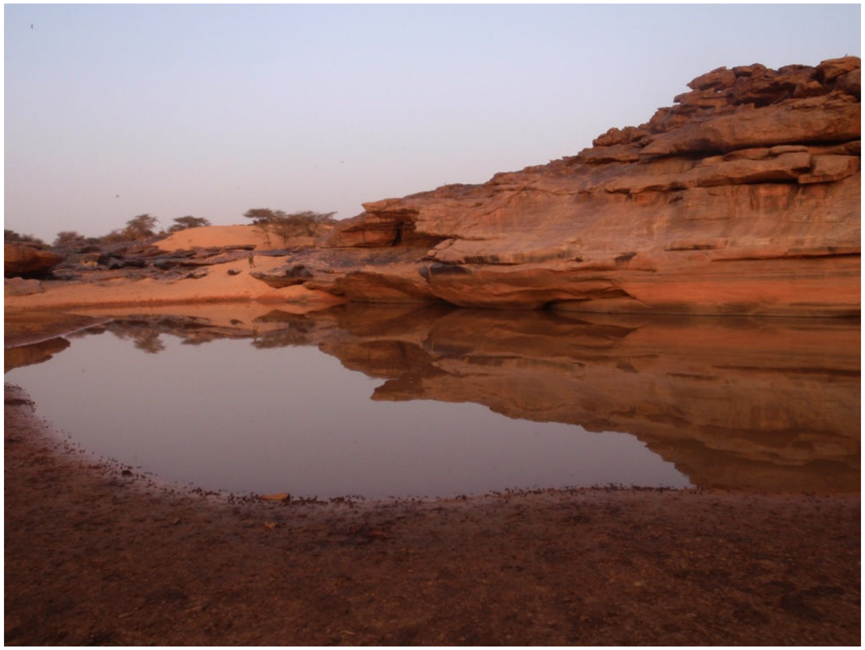

G03 Amzouzef
Photo: JC Brito

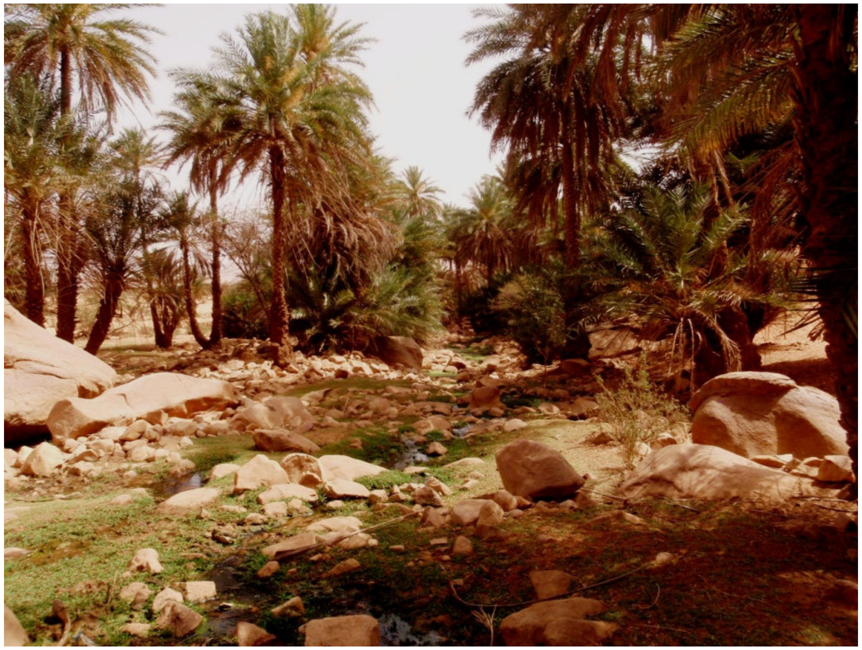

G04 Aouînet Nanâga
Extremely small *guelta*
Photo: JC Brito

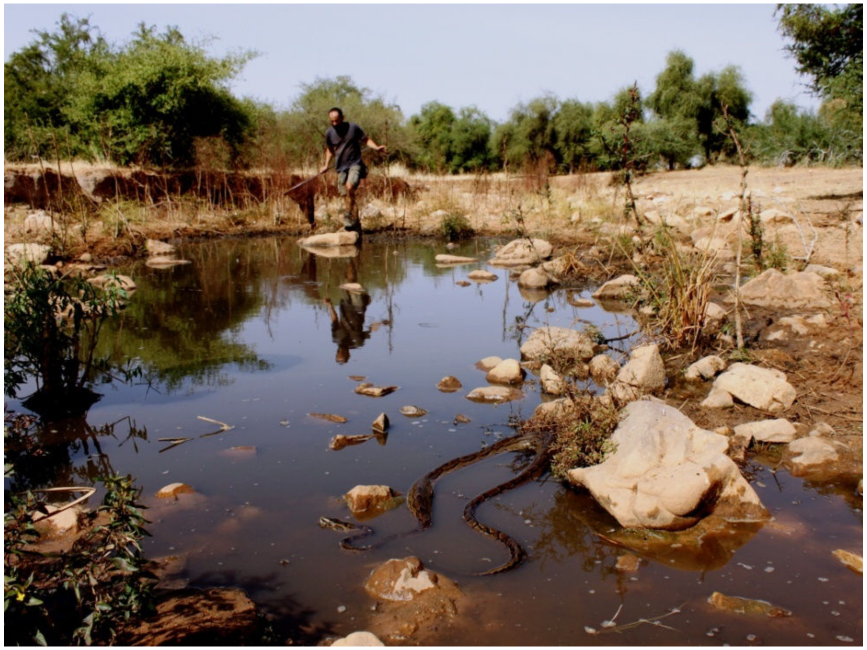

G06 Aouînet Tenbouckit
Python sebae in the foreground
Photo: JC Brito

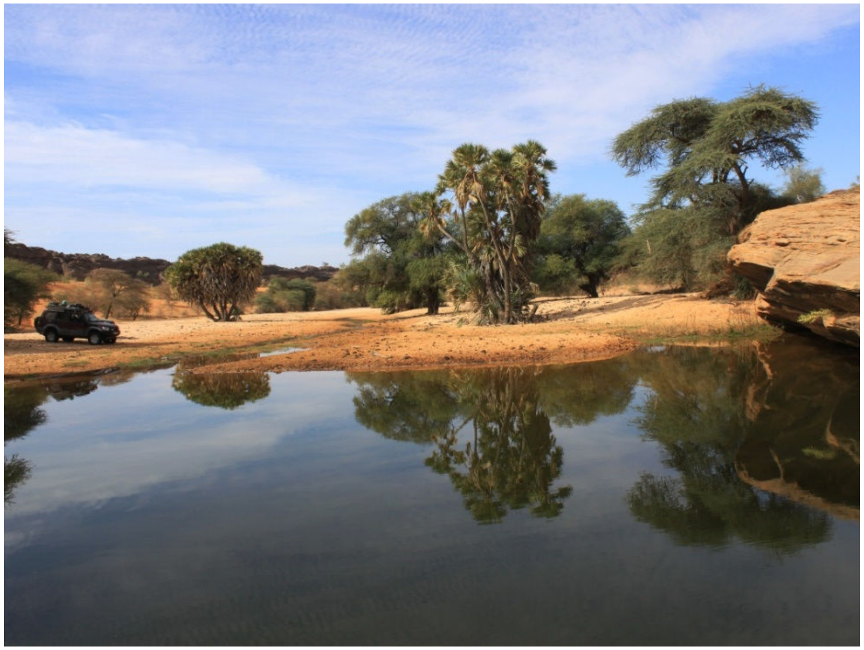

G07 Ayoûn en Na'aj
Photo: JC Brito

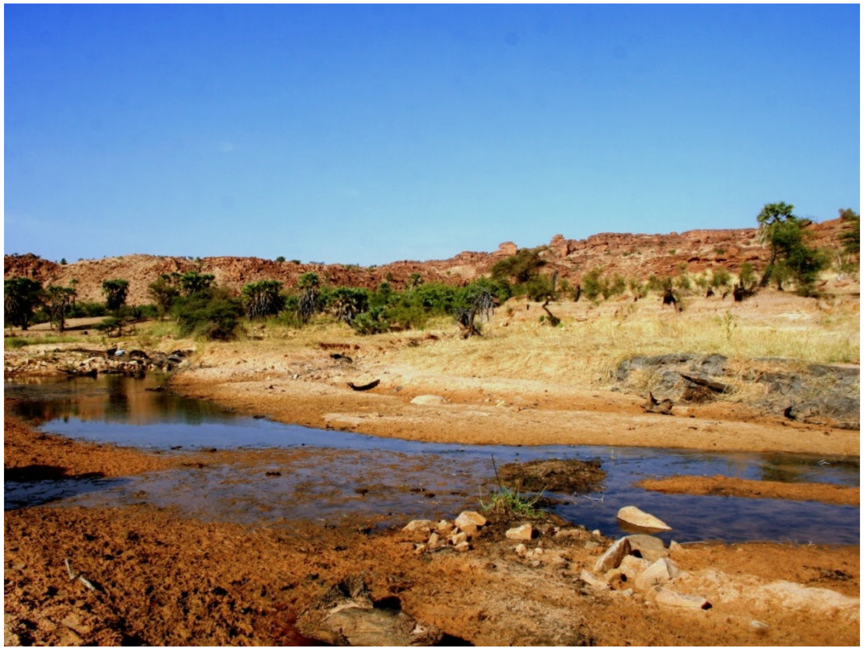

G08 Bâfa
Photo: F Martínez-Freiría

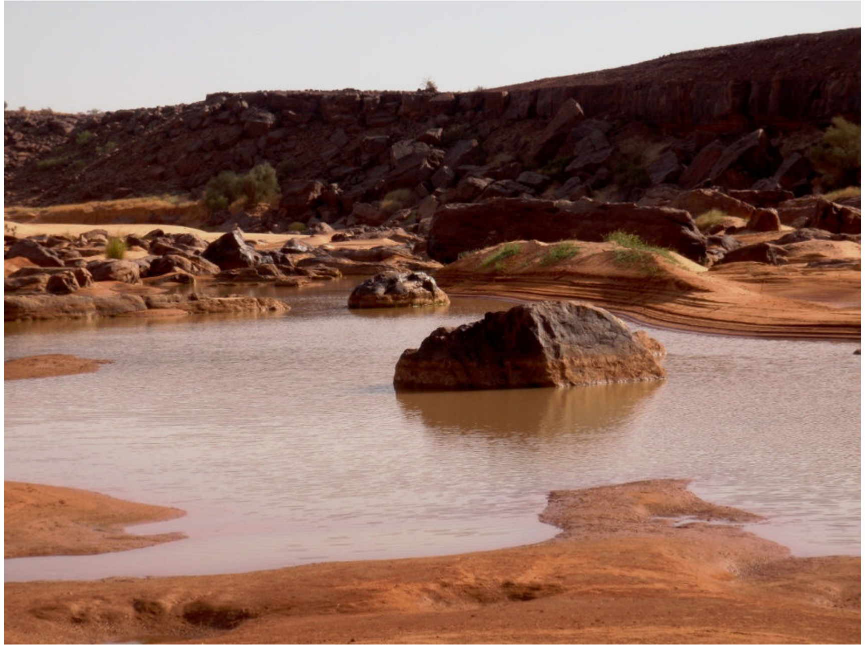

G09 Bajai
Photo: JC Brito

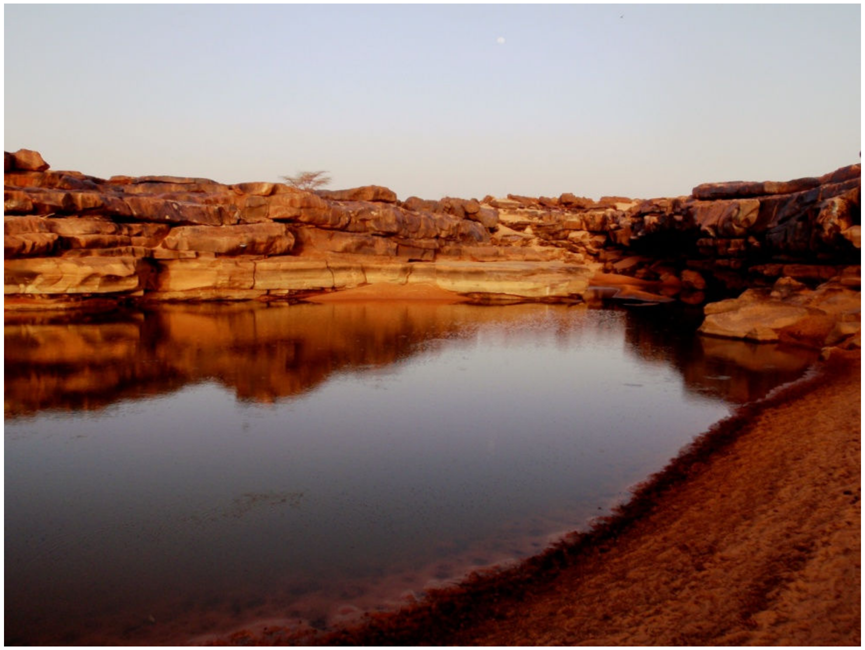

G10 Ch'Bayer
Photo: JC Brito

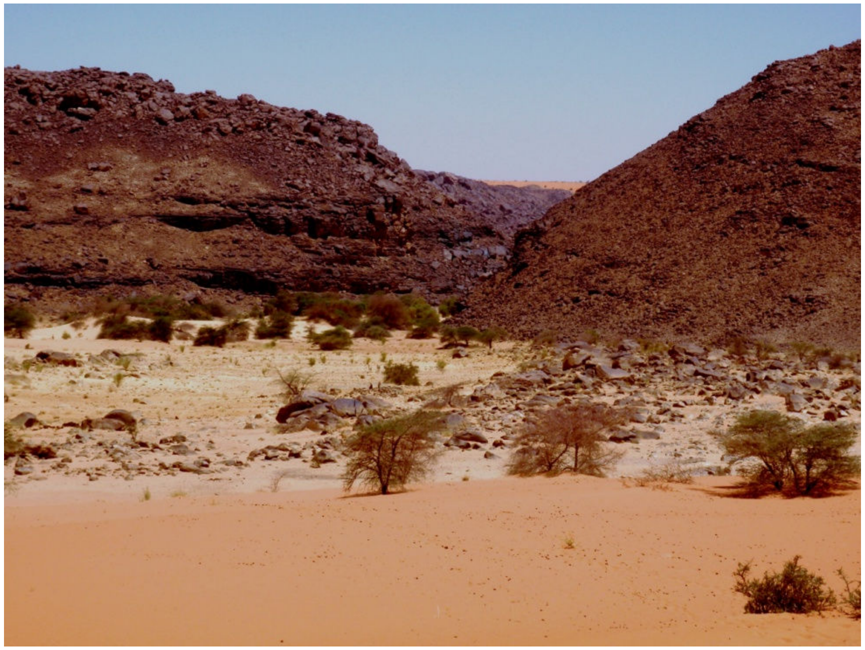

G11 Daal
Guelta (not observable) located in the narrow valley
Photo: JC Brito

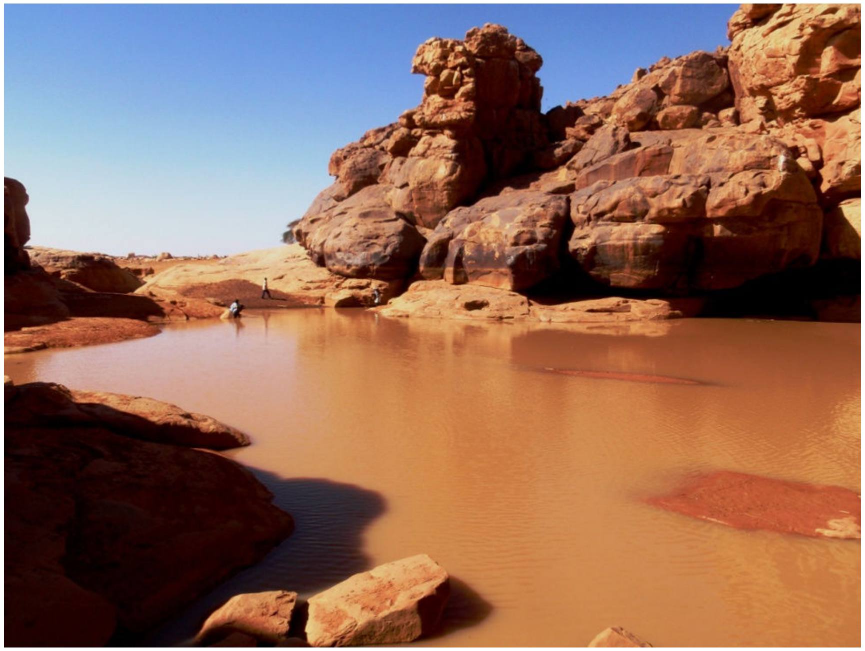

G12 Dâber
Photo: JC Brito

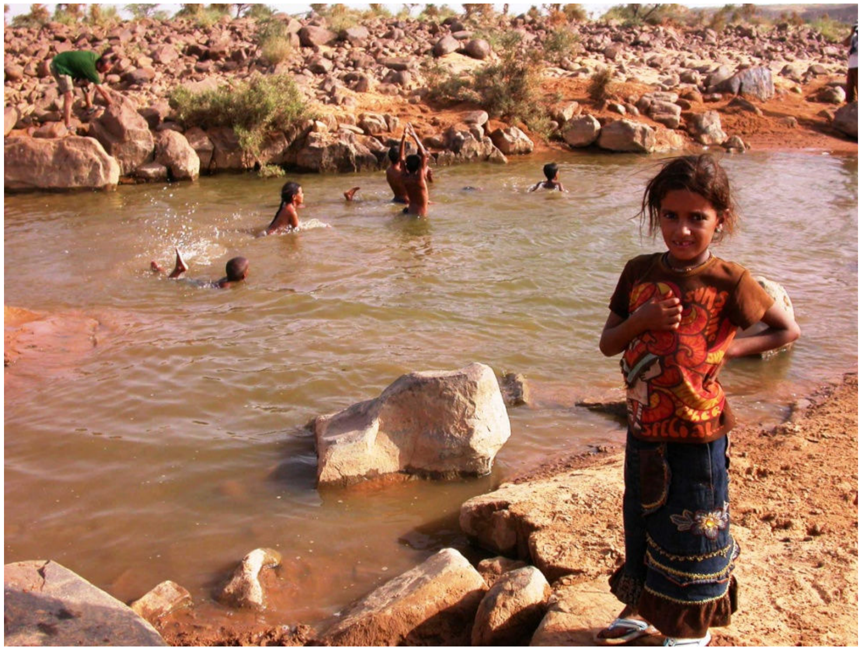

G13 Dâyet et Teila
Photo: F Martínez-Freiría

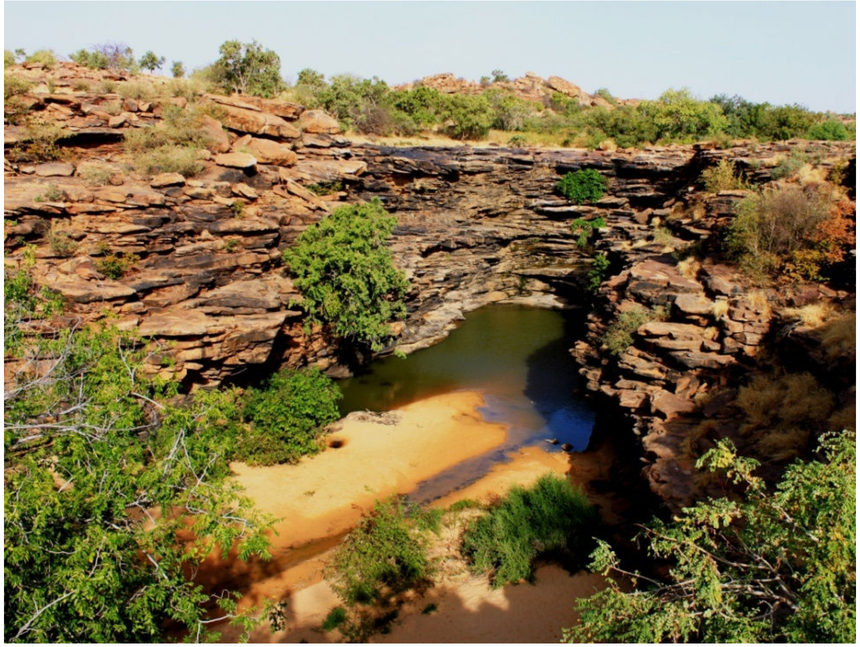

G15 El Barda
Photo: JC Brito

G16 El Ghâira
Photo: JC Brito

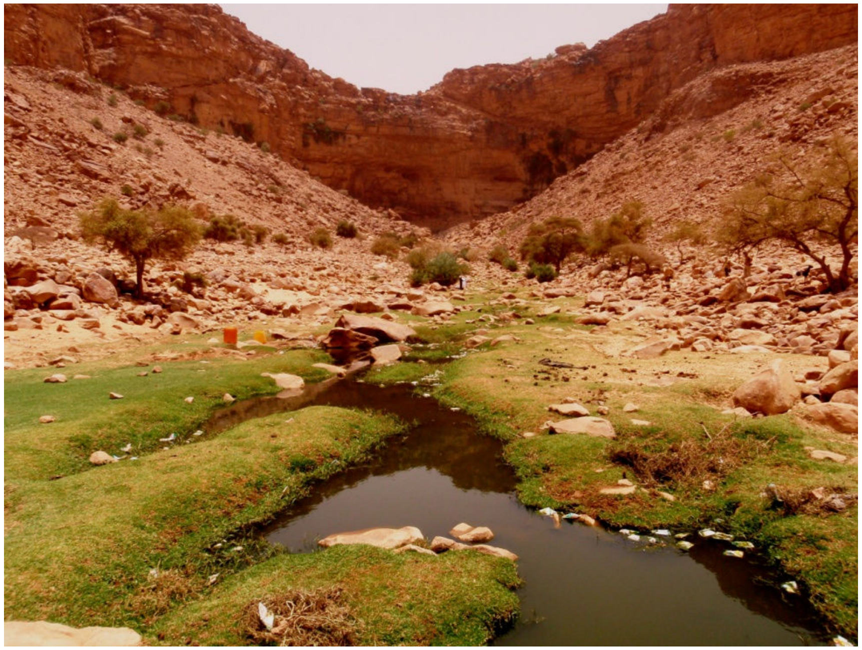

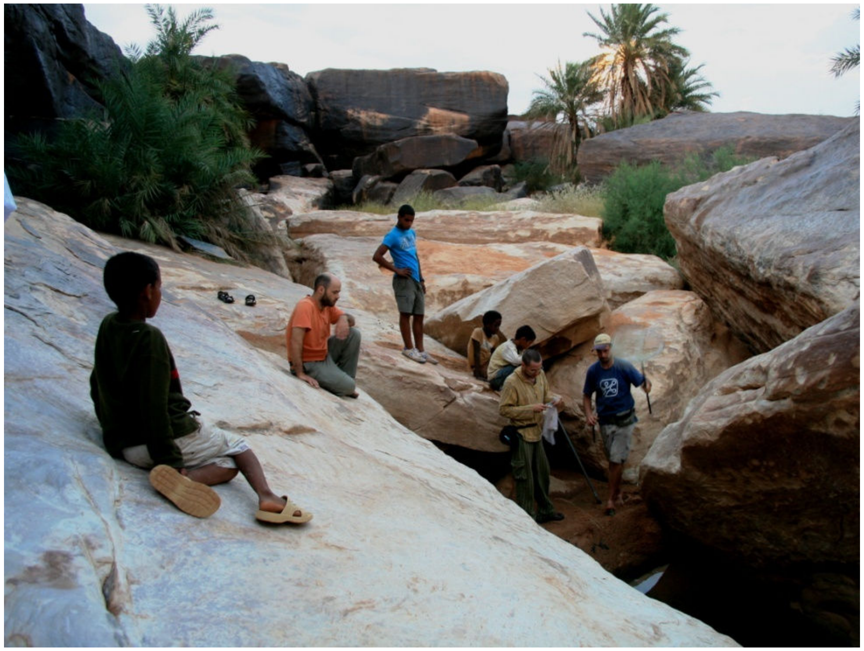

G17 El Gleitât
Sampling inside the nearly dry *guelta*
Photo: F Martínez-Freiría

G18 El Hnouk
Photo: F Martínez-Freiría

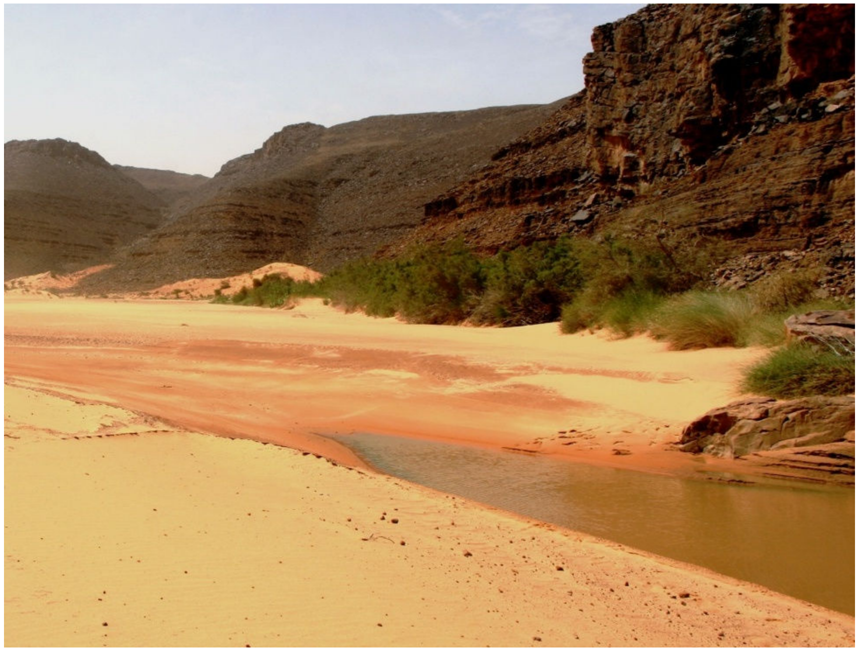

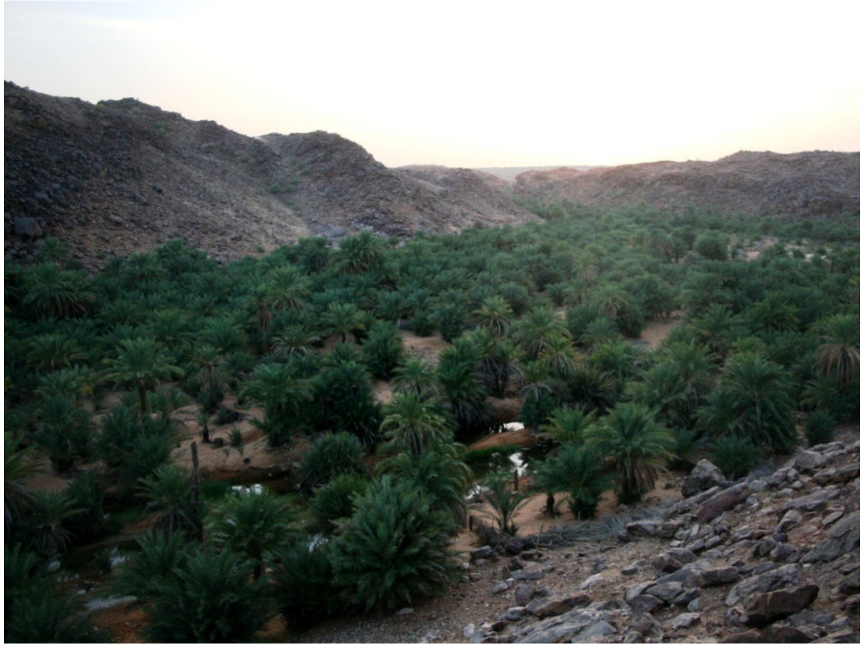

G19 El Housseînîya
Photo: F Martínez-Freiría

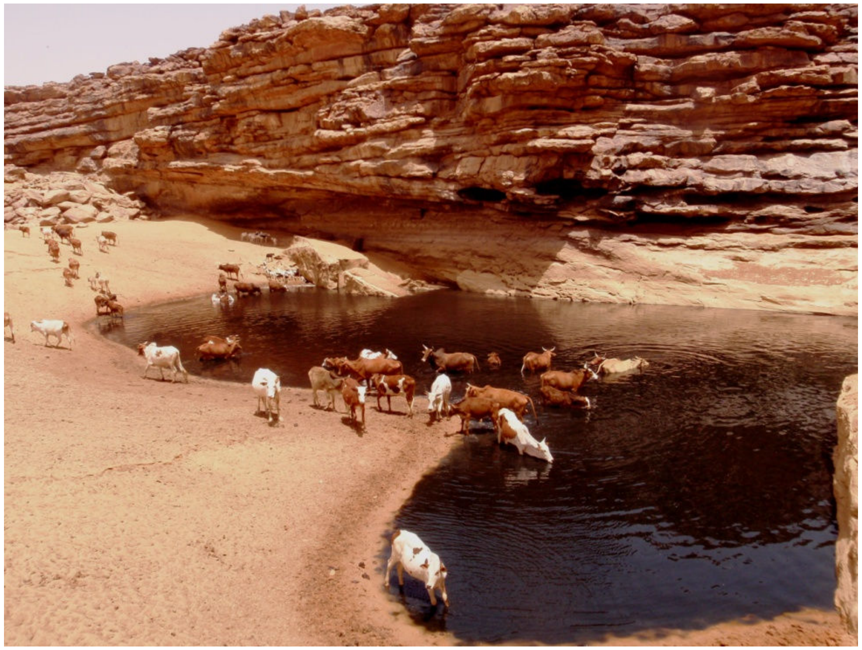

G20 El Khedia
Photo: JC Brito

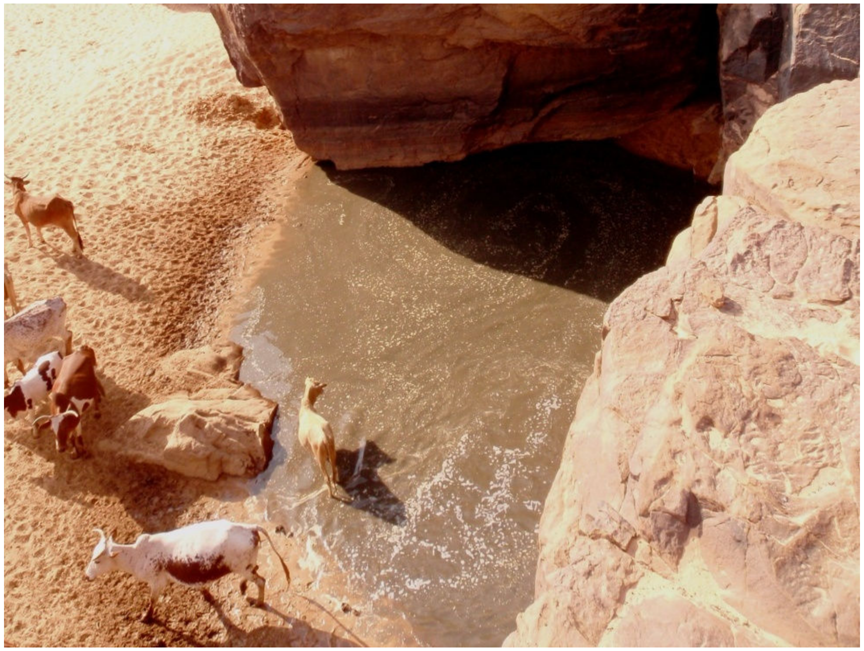

G21 El Mefga
Photo: JC Brito

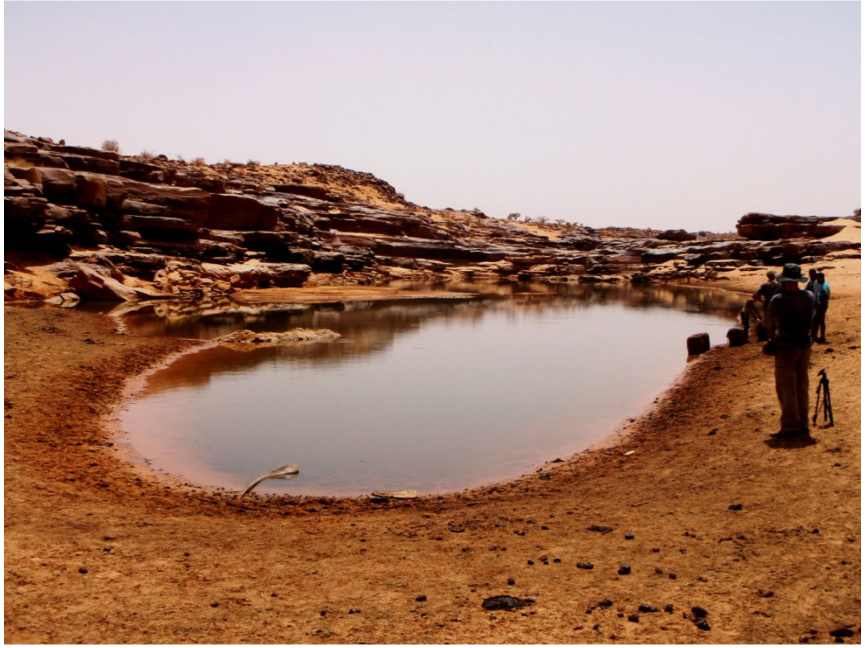

G22 Emreimida
Photo: N Sillero

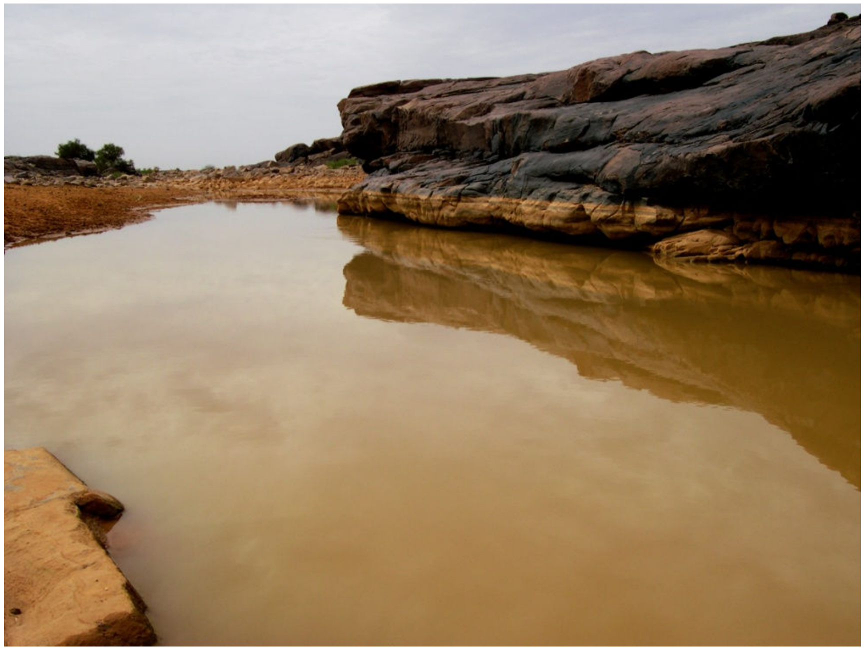

G23 E-n-Guinâr
Photo: JC Brito

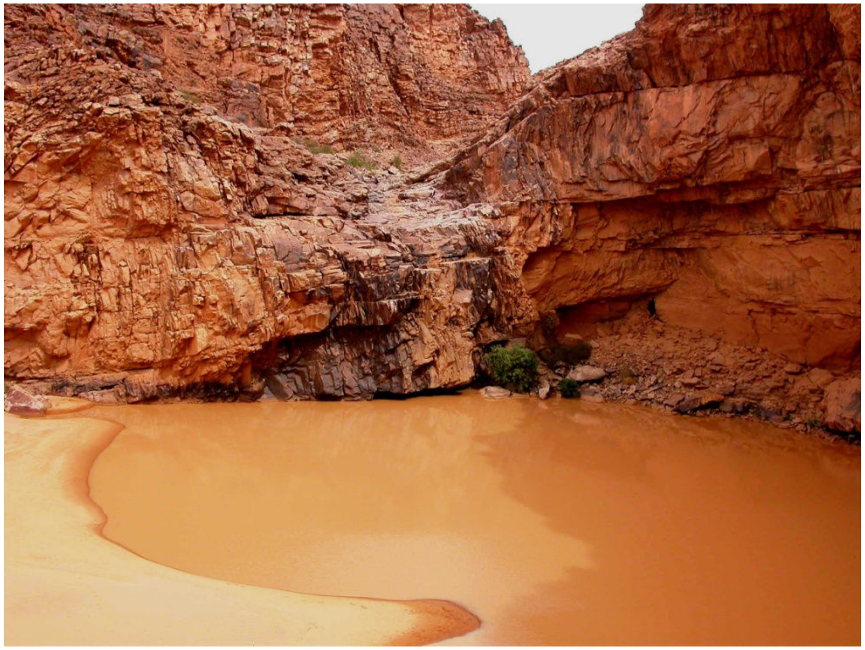

G25 Foum el Kour
Photo: F Martínez-Freiría

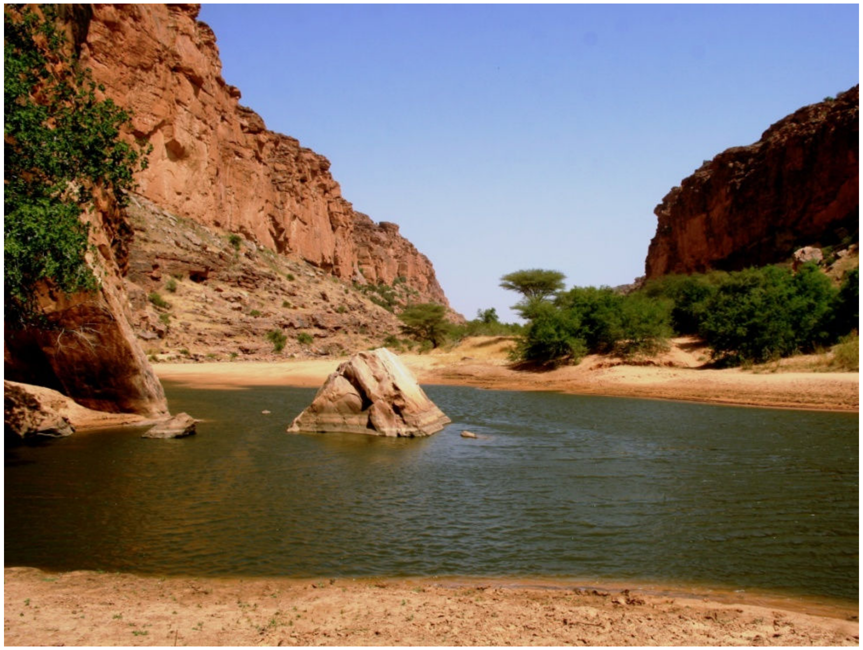

G26 Foum Goussas
Photo: F Martínez-Freiría

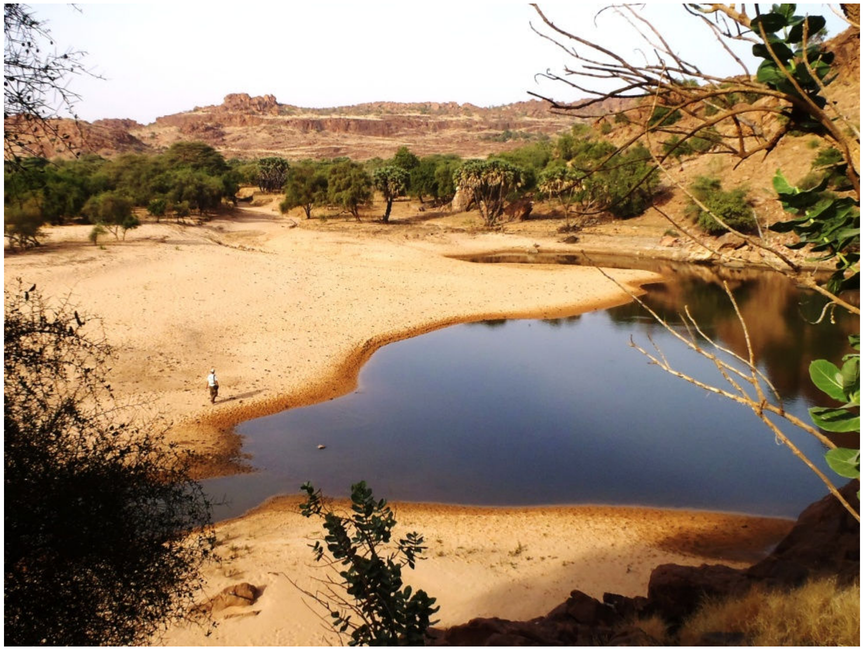

G27 Galoûla
Photo: Z Boratyński

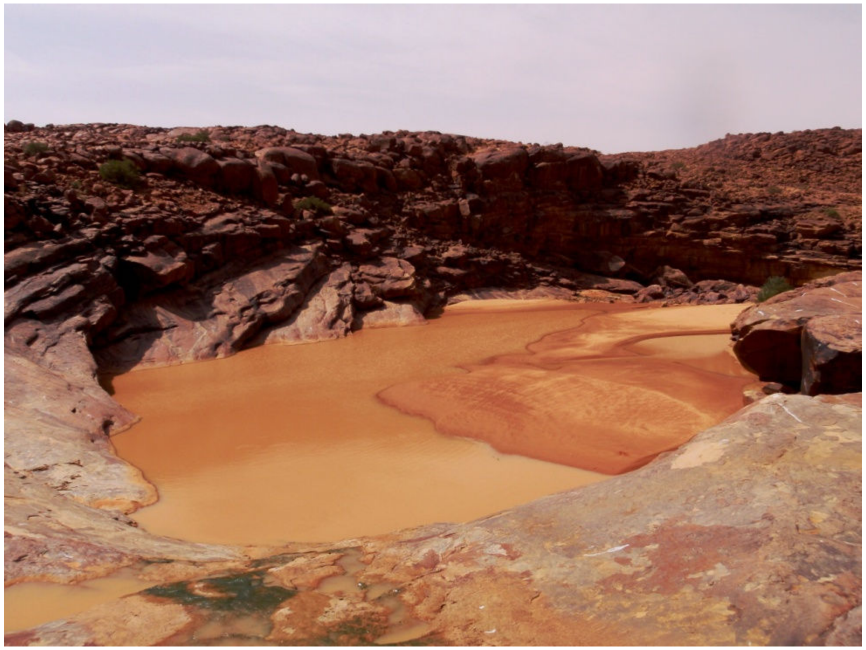

G28 Gamra Ouarbî
Photo: JC Brito

G29 Gânçai
Extremely small *guelta* (not observable) surrounded by dense vegetation
Photo: F Martínez-Freiría

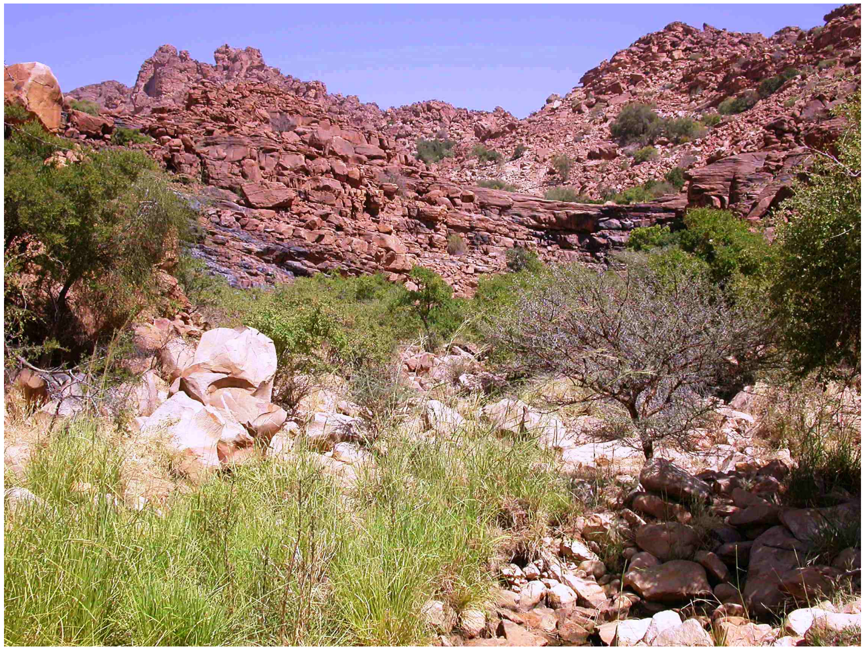

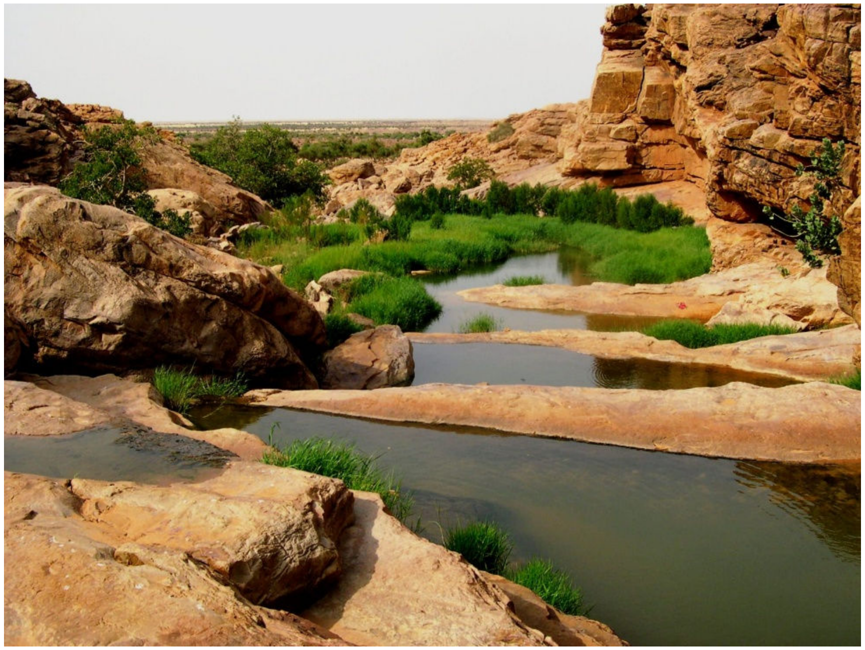

G30 Garaouel
Photo: JC Brito

G31 Glât el Bil
Photo: JC Brito

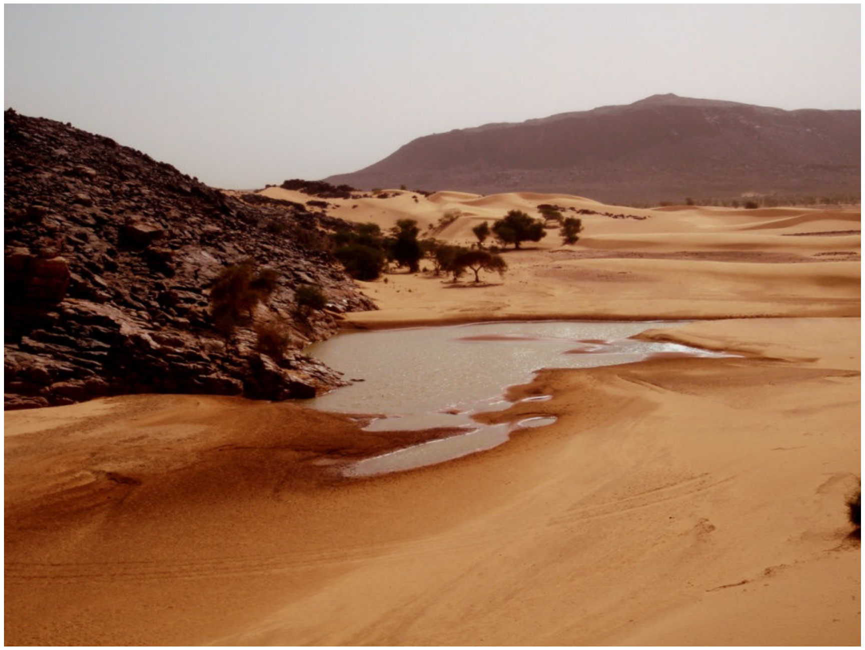

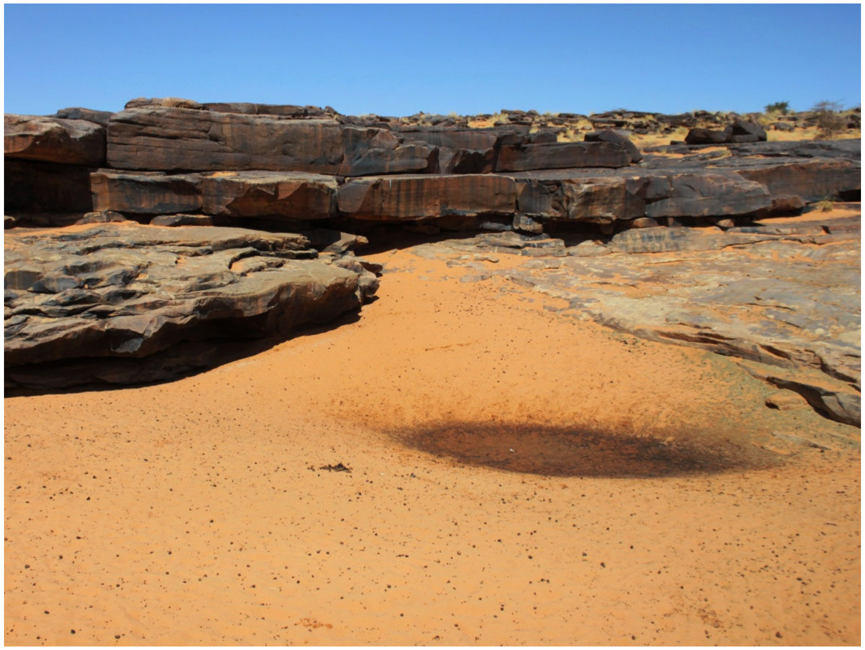

G32 Gleitat Ej Jmel
Guelta was nearly dry at time of visit
Photo: JC Brito

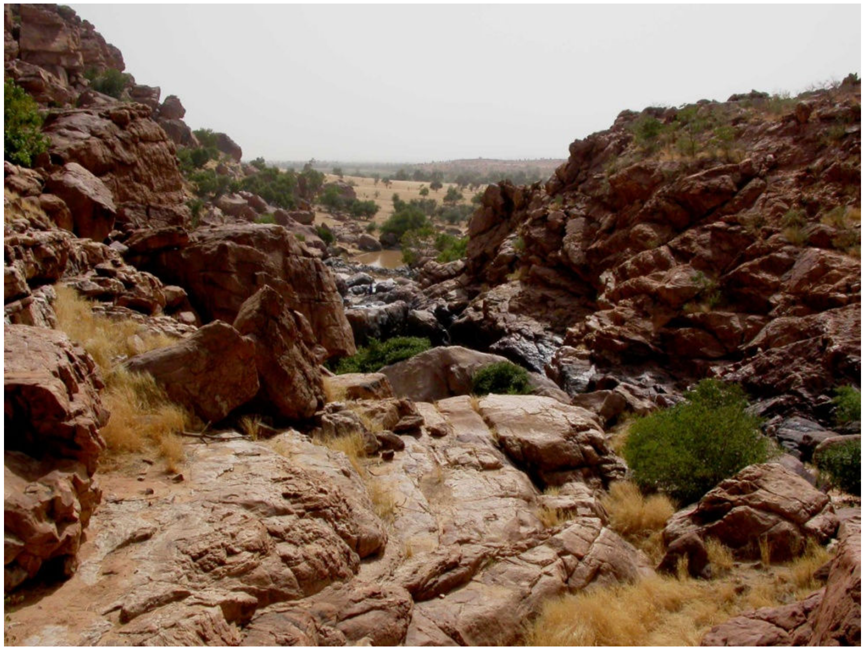

G33 Goumbel
Photo: F Martínez-Freiría

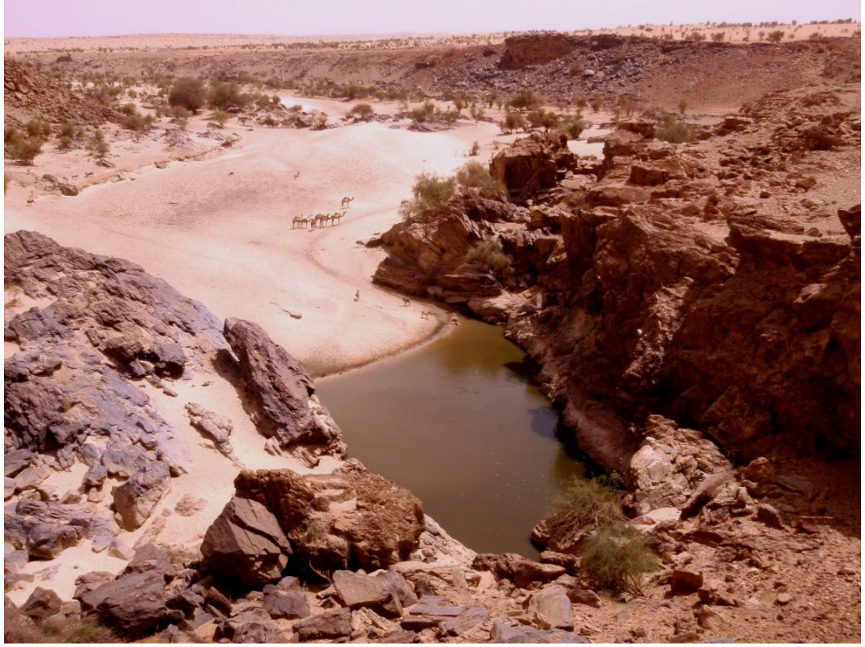

G34 Gueltet Thor
Photo: JC Brito

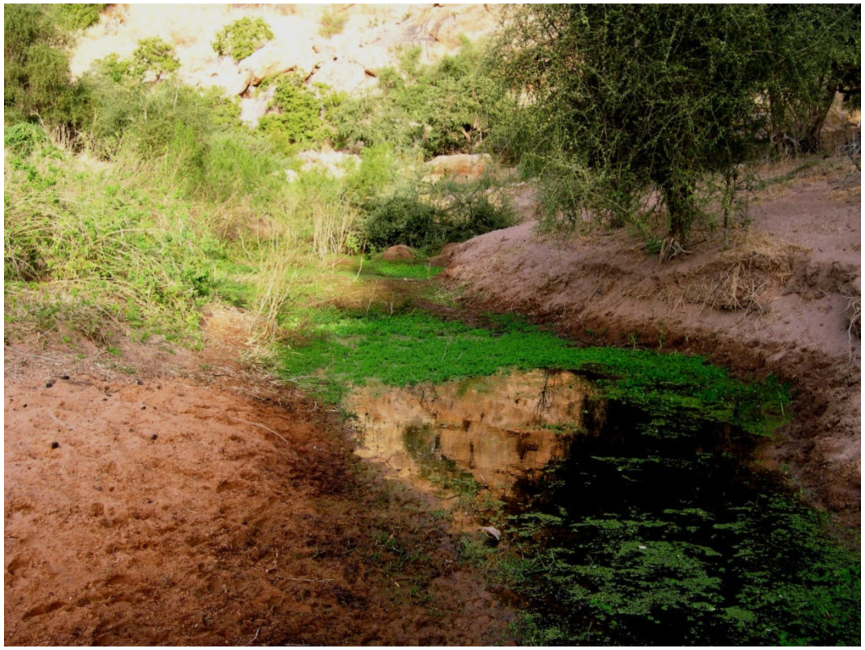

G35 Guenétir
Photo: JC Brito

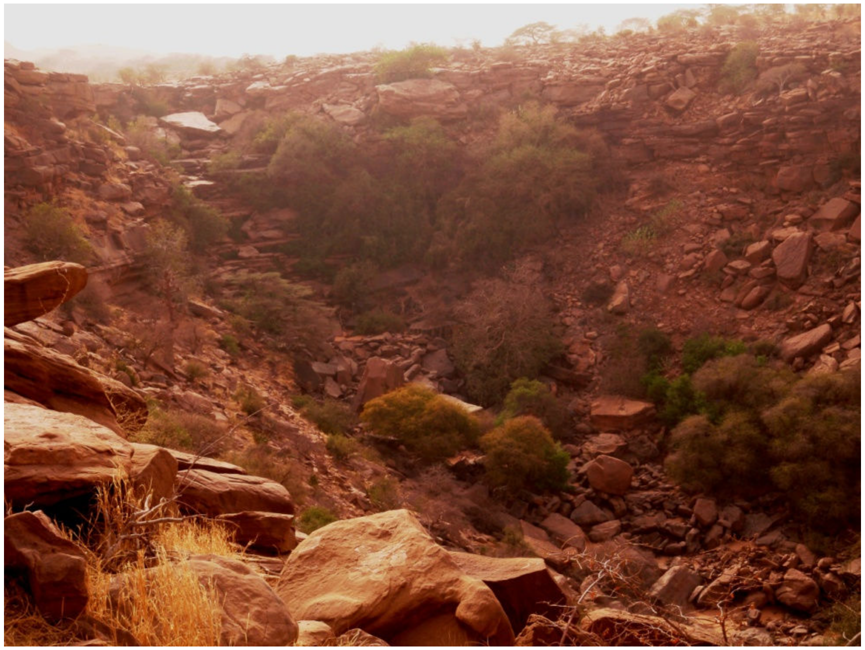

G36 Guérou
Guelta (not observable) is located in the bottom of the valley
Photo: JC Brito

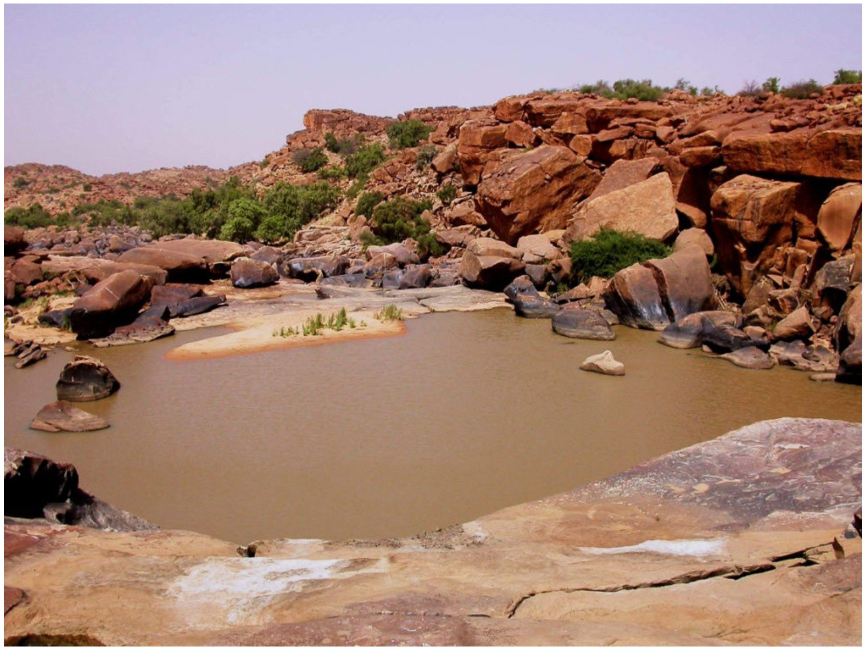

G37 Guidemballa
Photo: F Martínez-Freiría

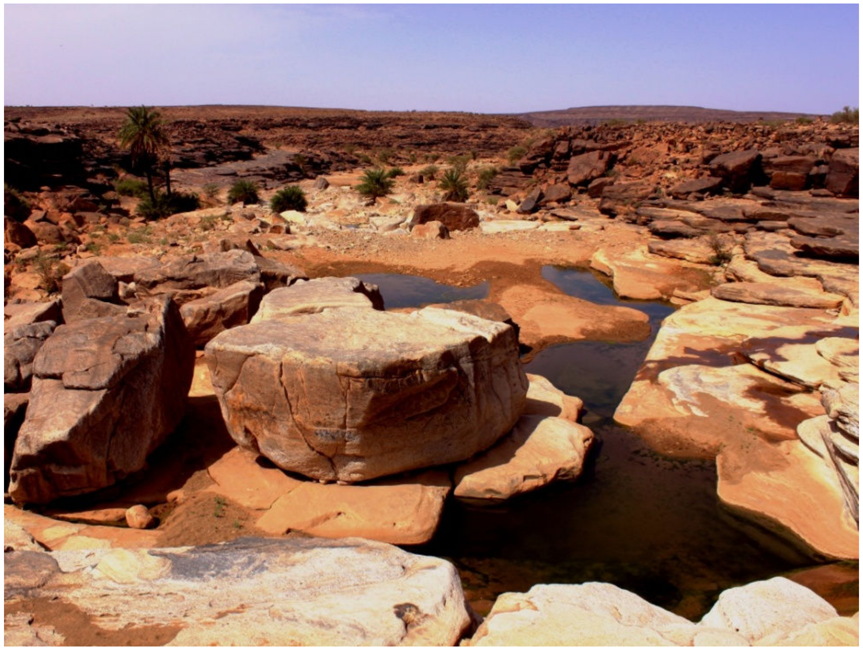

G38 Hamdoûn
Photo: JC Brito

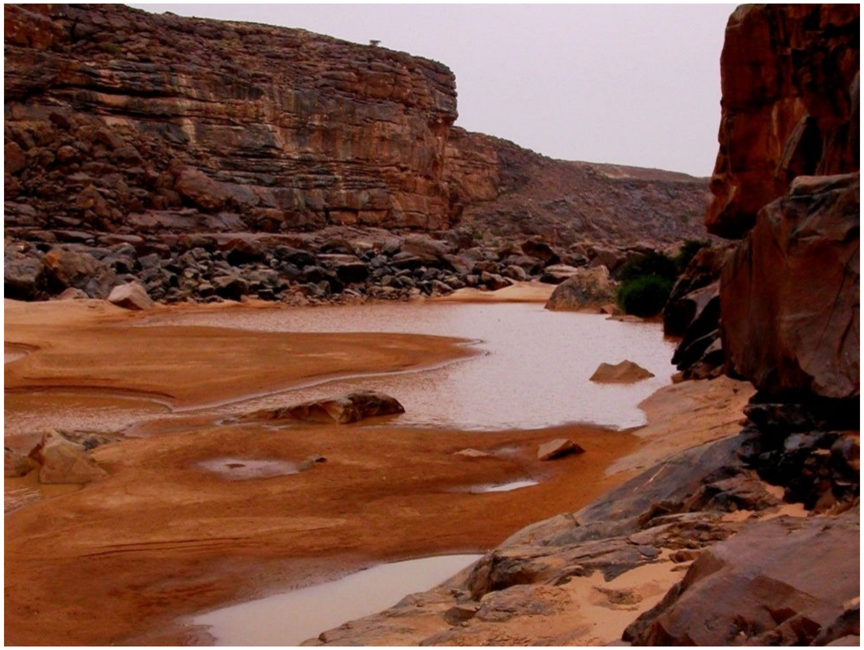

G39 Jabara
Photo: F Martínez-Freiría

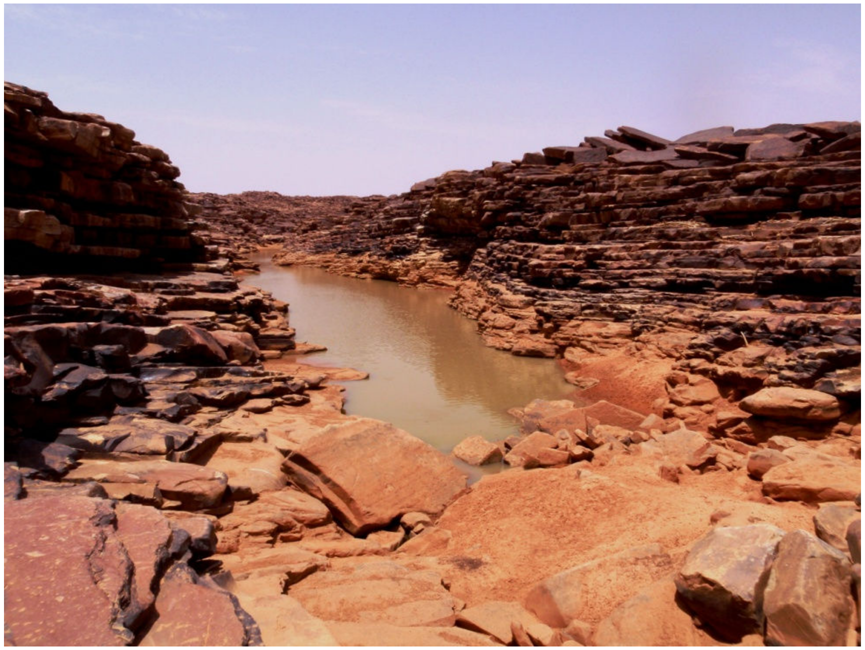

G40 Kabda
Photo: JC Brito.

G42 El Grâne
Guelta (not observable) is located inside the canyon in front
Photo: Z Boratyński.

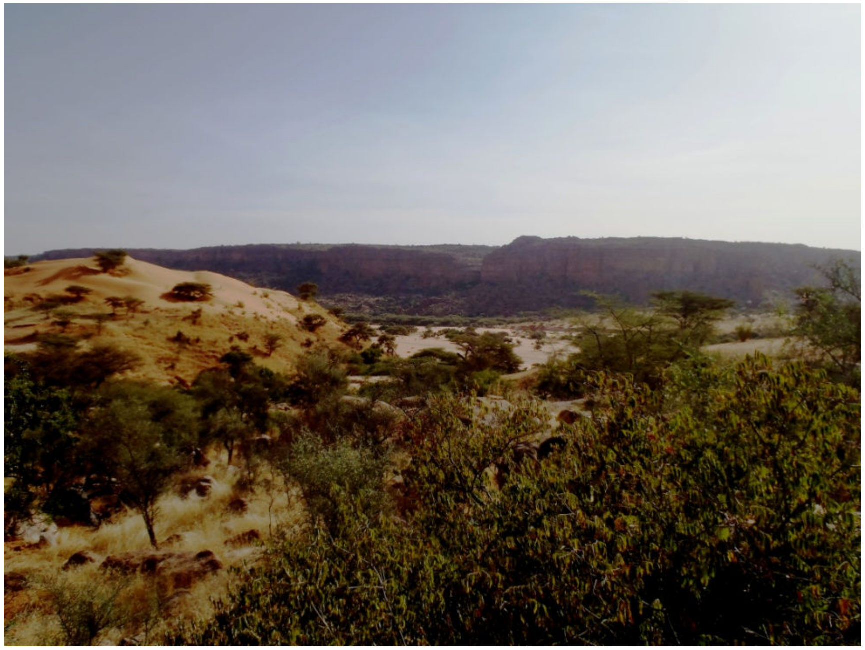

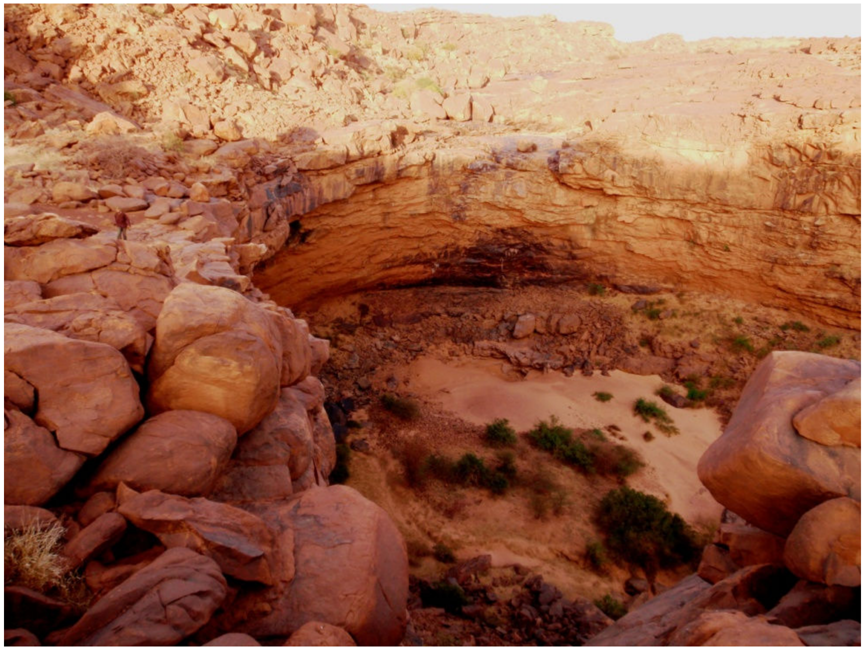

G43 Laout
Photo: JC Brito

G44 Laout, 1km S of
Photo: JC Brito

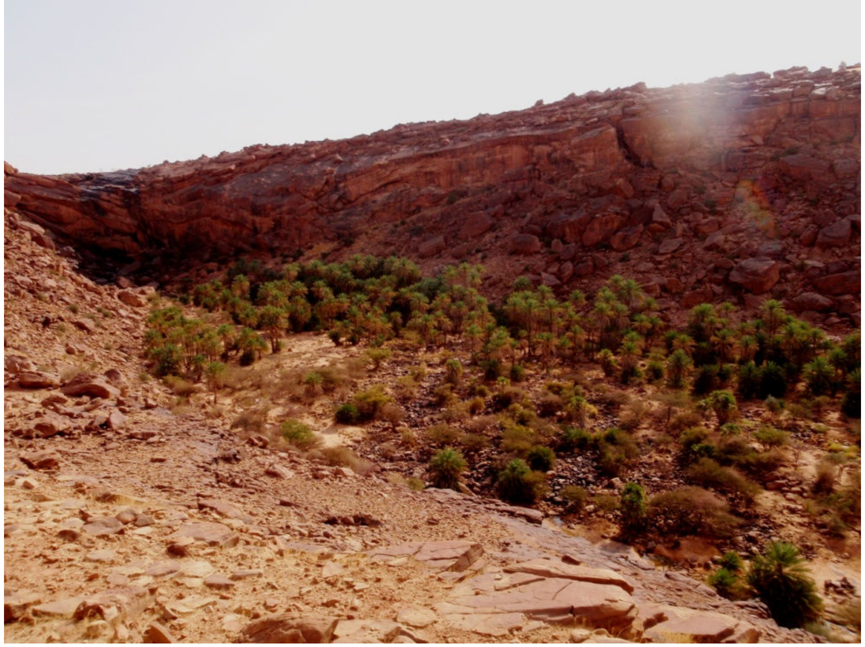

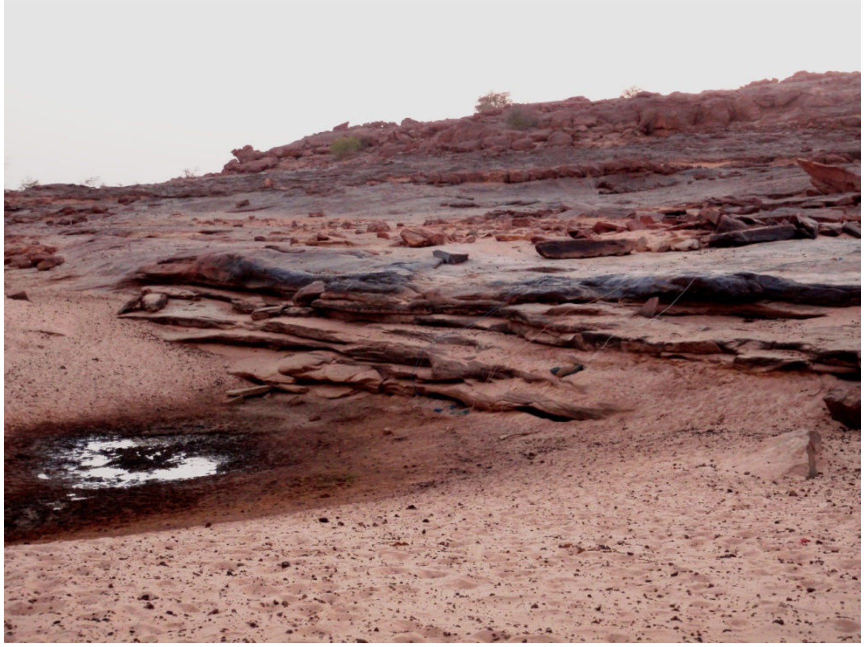

G45 Legleyta
Guelta was nearly dry at time of visit
Photo: JC Brito

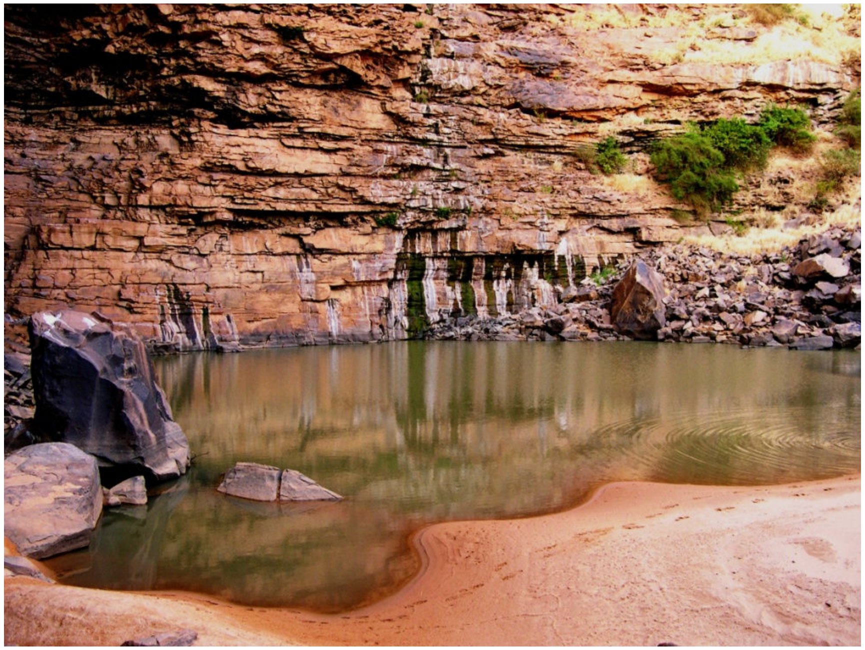

G46 Lemmollah
Photo: JC Brito

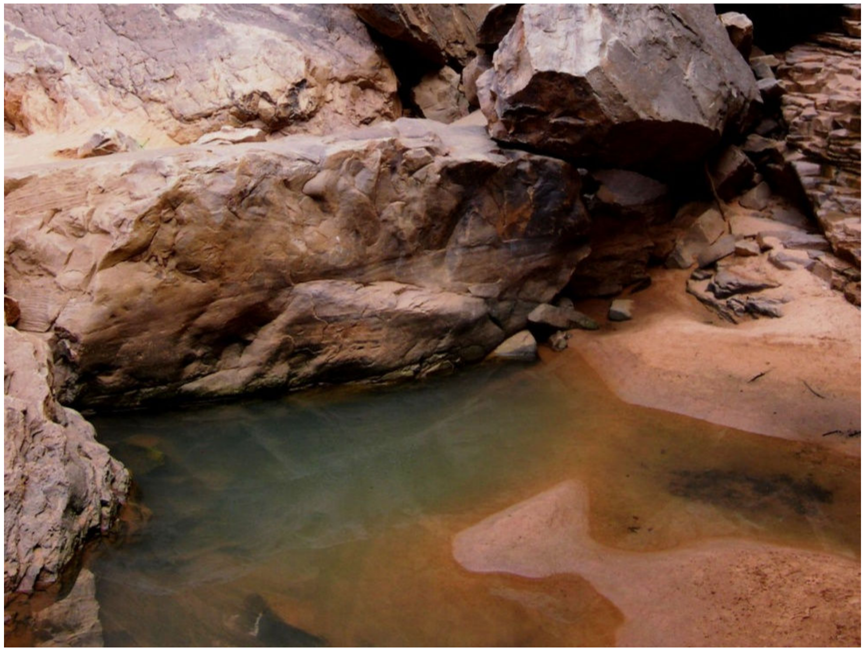

G47 Leouel
Photo: JC Brito

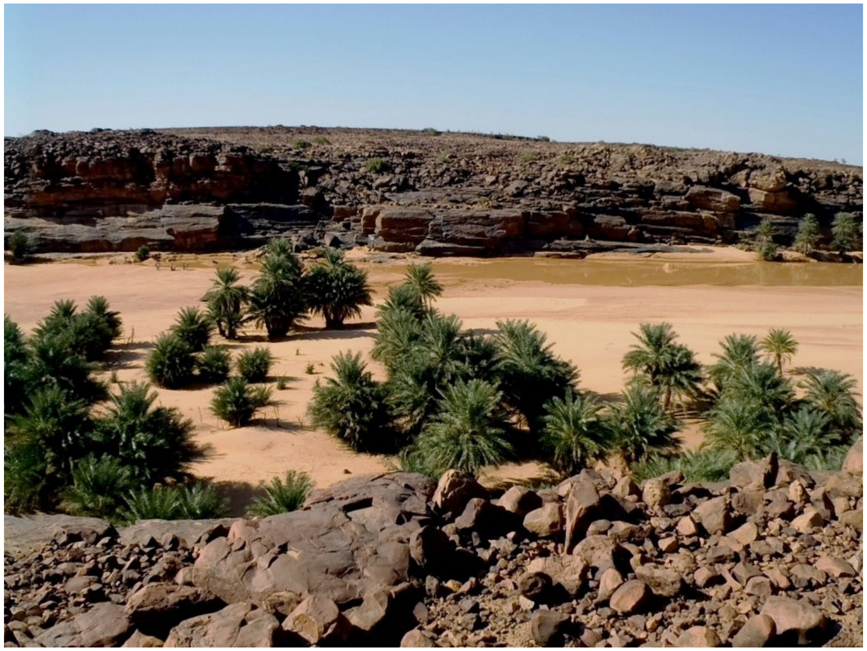

G48 Matmâta
Photo: Z Boratyński

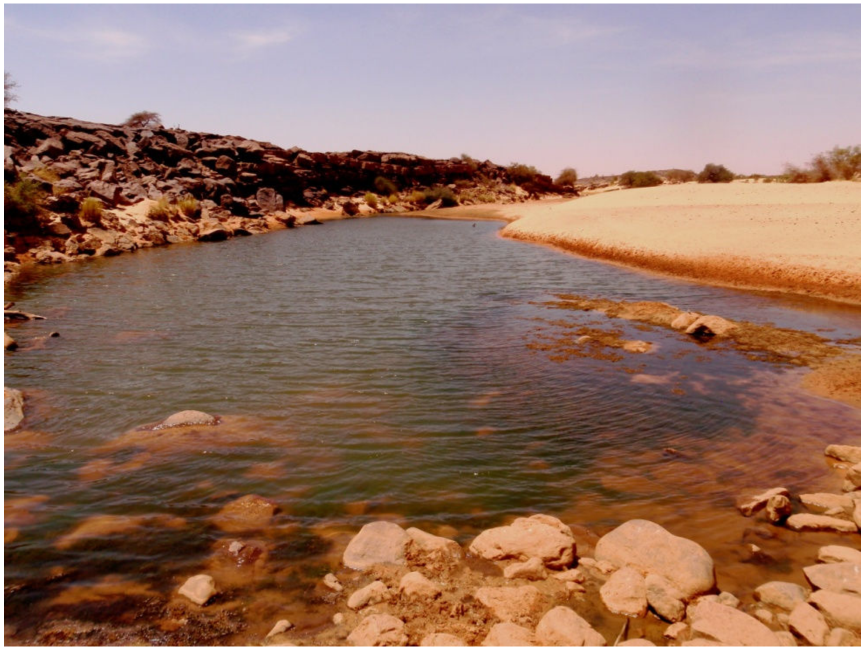

G49 M'cherba
Photo: JC Brito

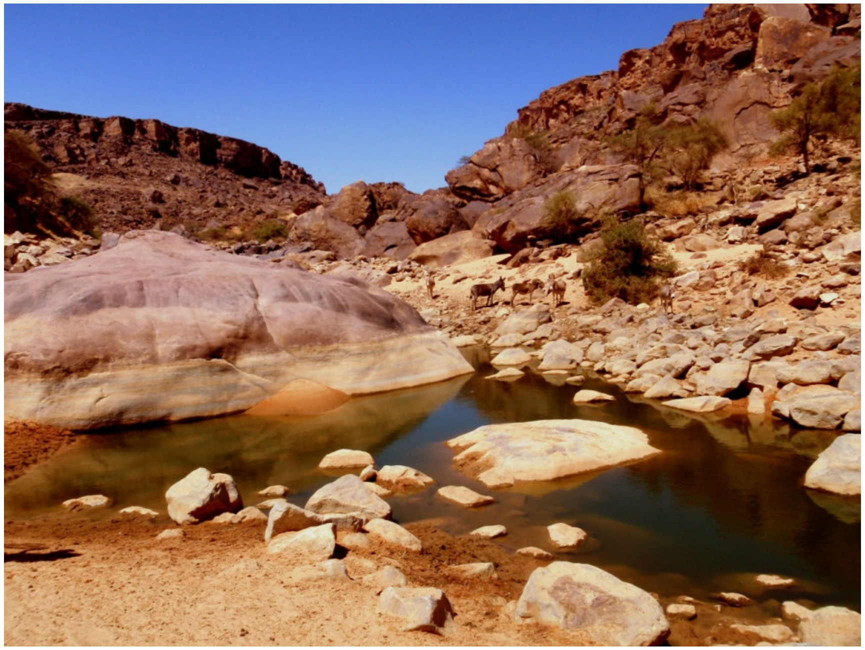

G50 Mendjoura
Photo: JC Brito

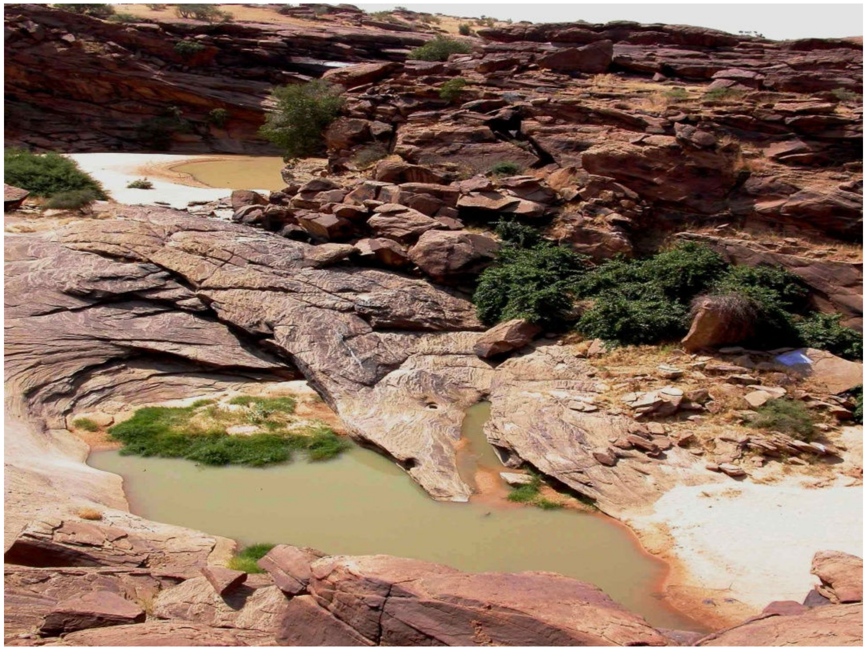

G51 Metraoucha
Photo: F Martínez-Freiría

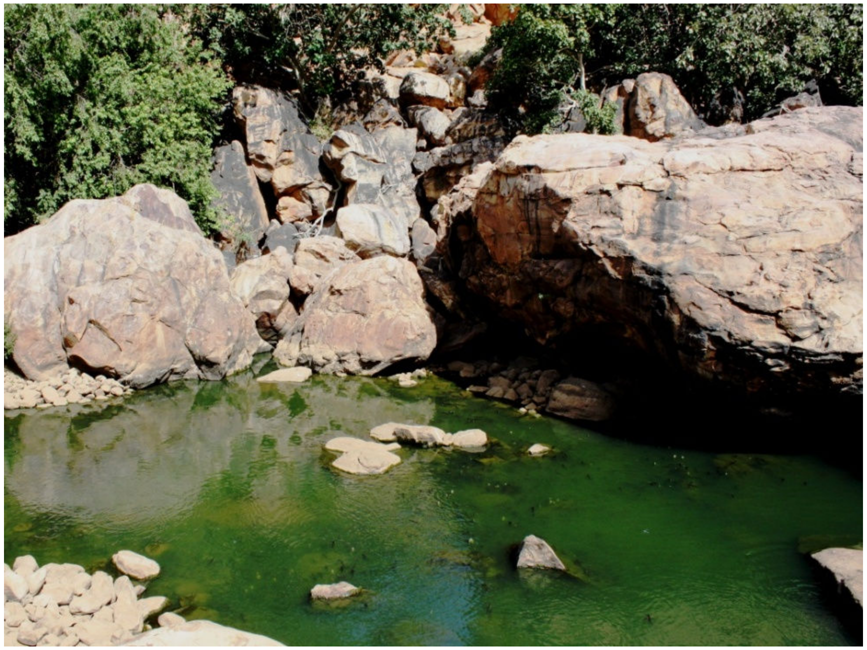

G52 Meyla
Photo: JC Brito

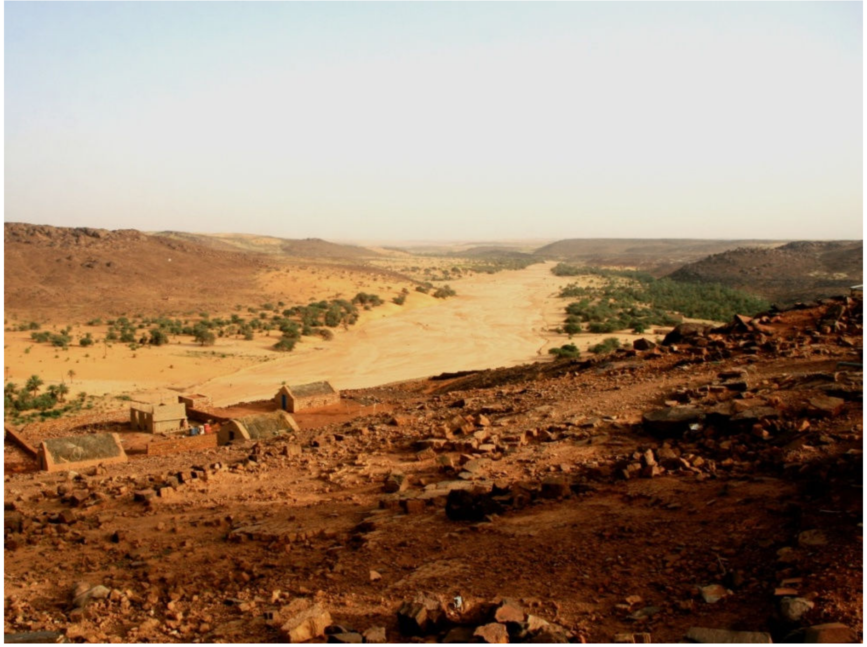

G53 Oumm el Arjam
Guelta (not observable) is located at right, close to the dense palm trees
Photo: F Martínez-Freiría

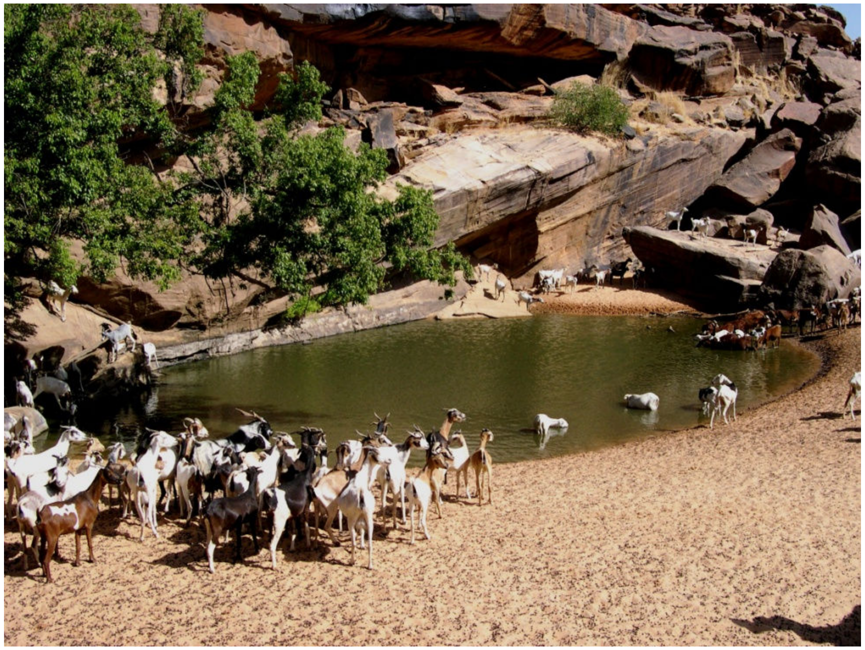

G54 Oumm el Mhâr
Photo: JC Brito

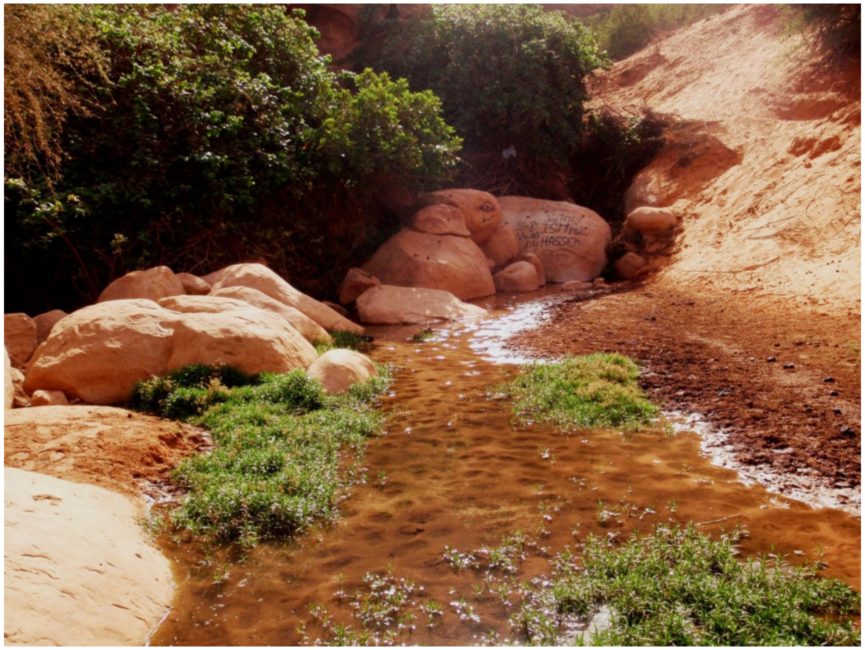

G55 Oumm Icheglâne
Photo: JC Brito

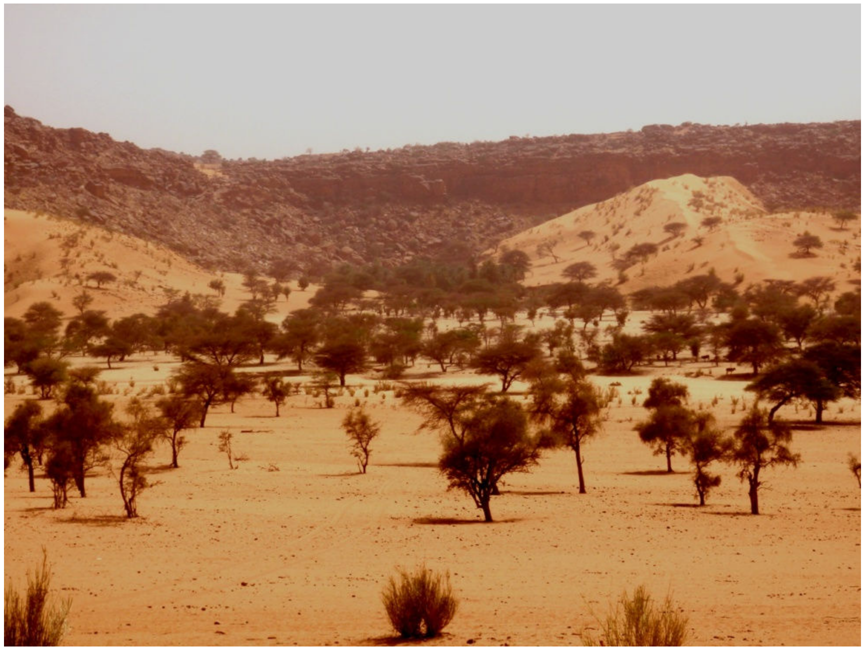

G56 Oumm Icheglâne, 5km NW of
Guelta (not observable) is located in bottom of the valley
Photo: JC Brito

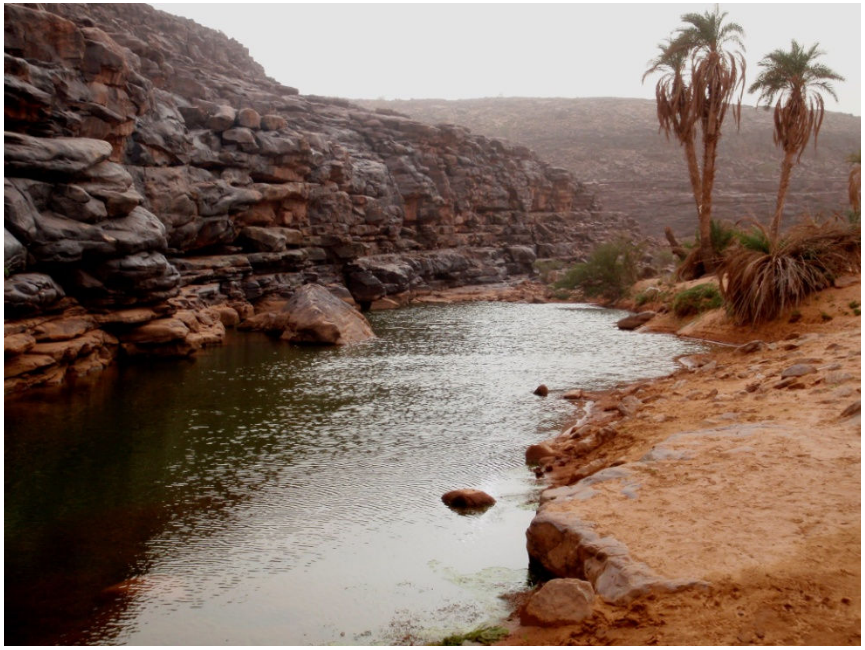

G57 Oumm Lemhâr
Photo: JC Brito

G58 Rh' Zembou
Photo: JC Brito

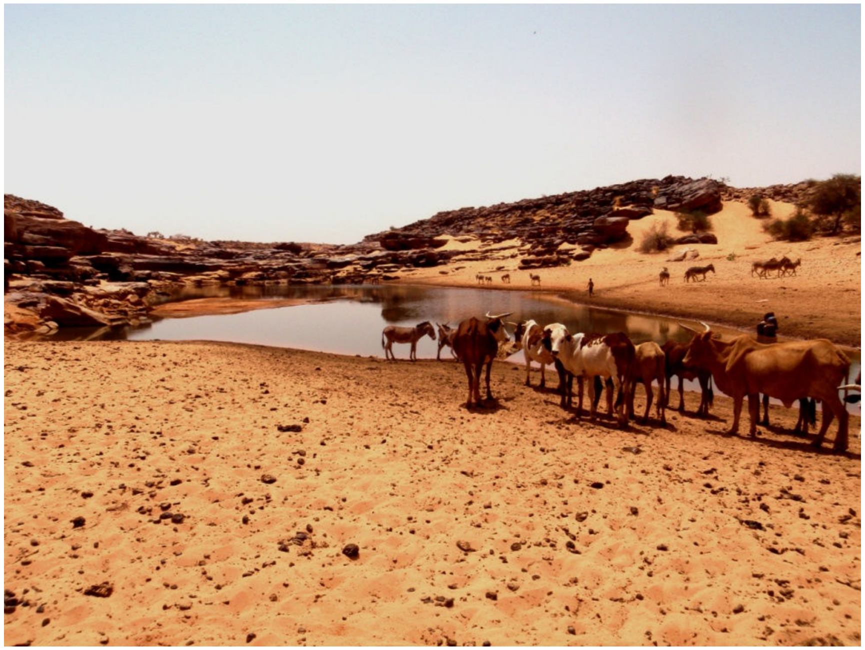

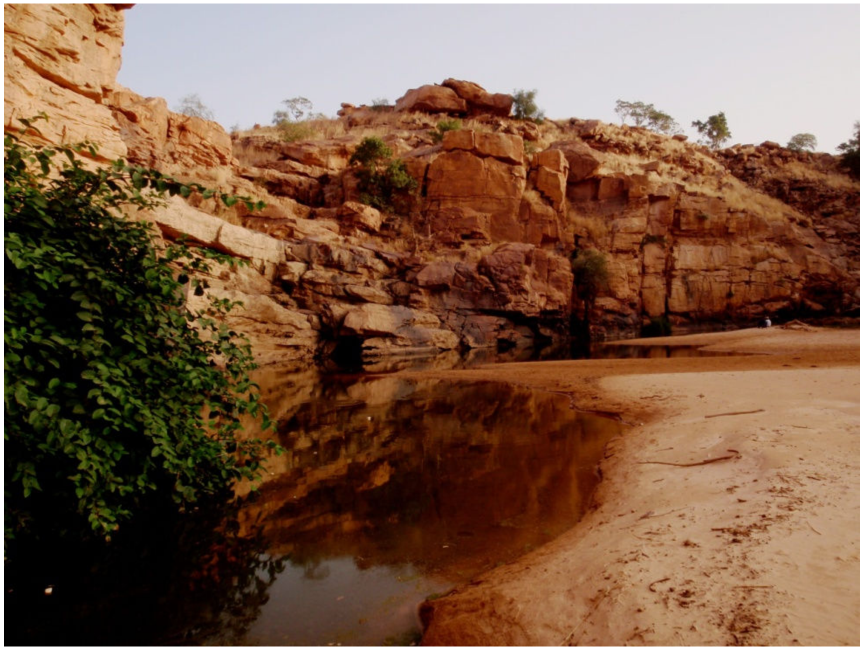

G59 Soufa
Photo: JC Brito

G61 Taorta
Photo: JC Brito

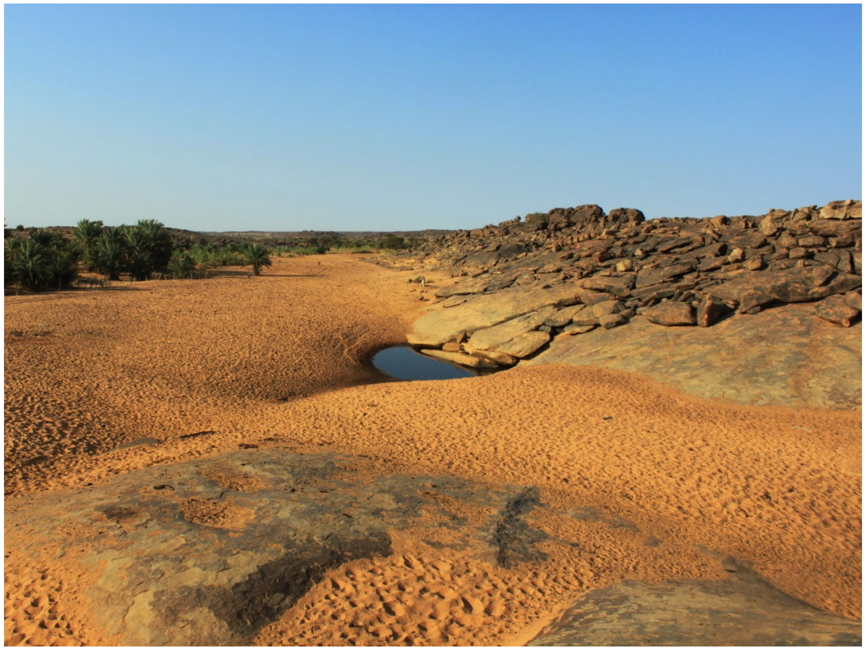

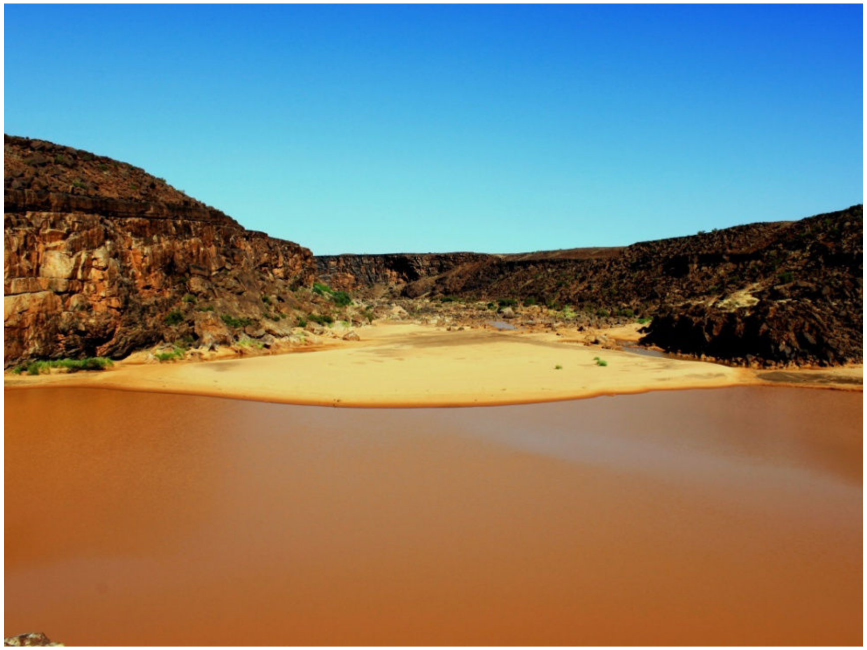

G62 Tartêga
Photo: JC Brito

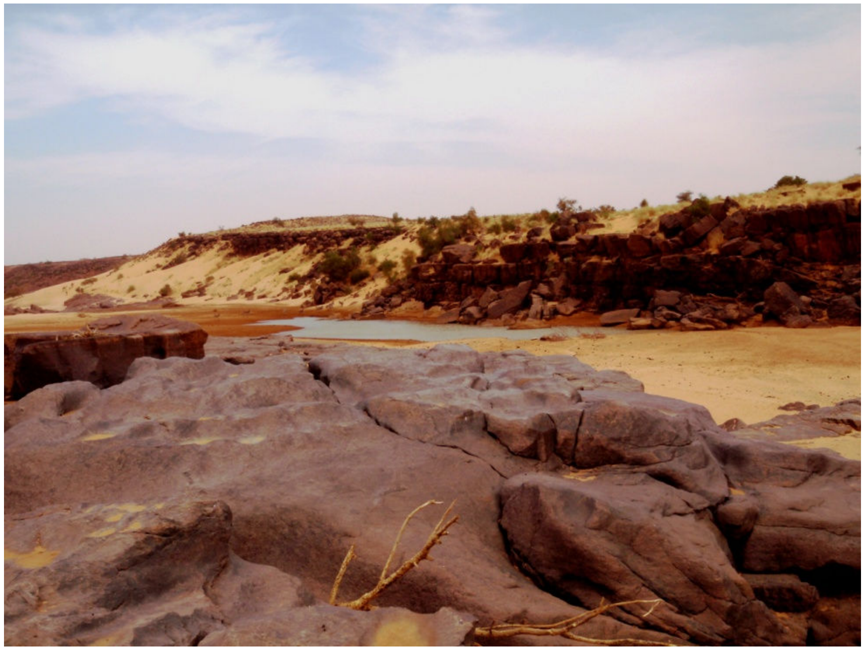

G63 Tartêga, upstream of
Photo: JC Brito

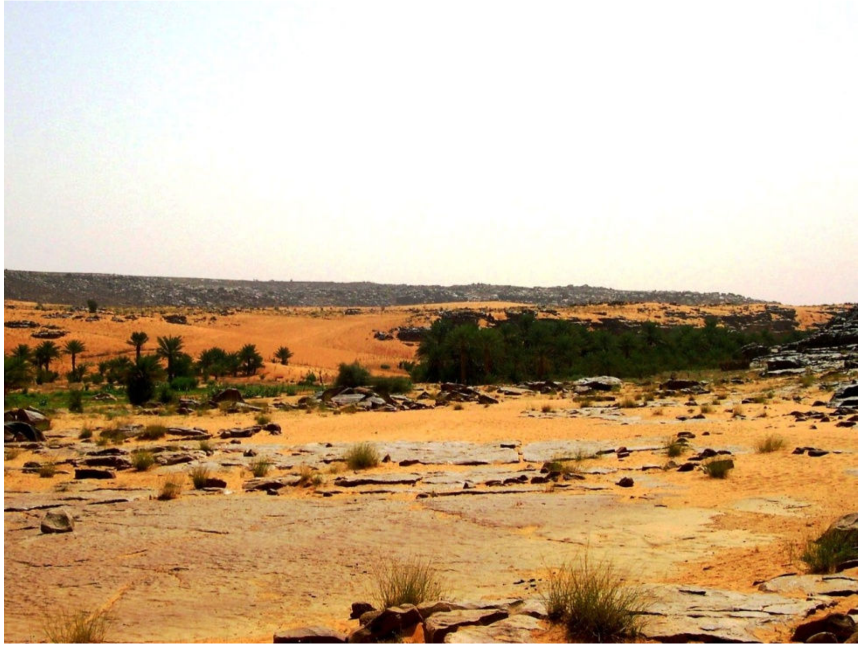

G64 Taoujafet
Photo: JC Brito

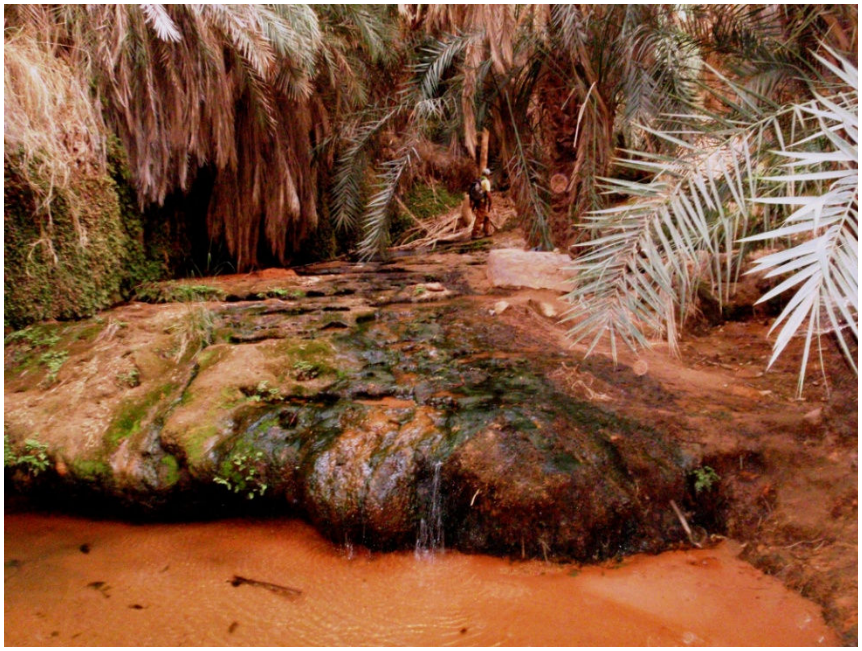

G65 Terjît
Photo: JC Brito

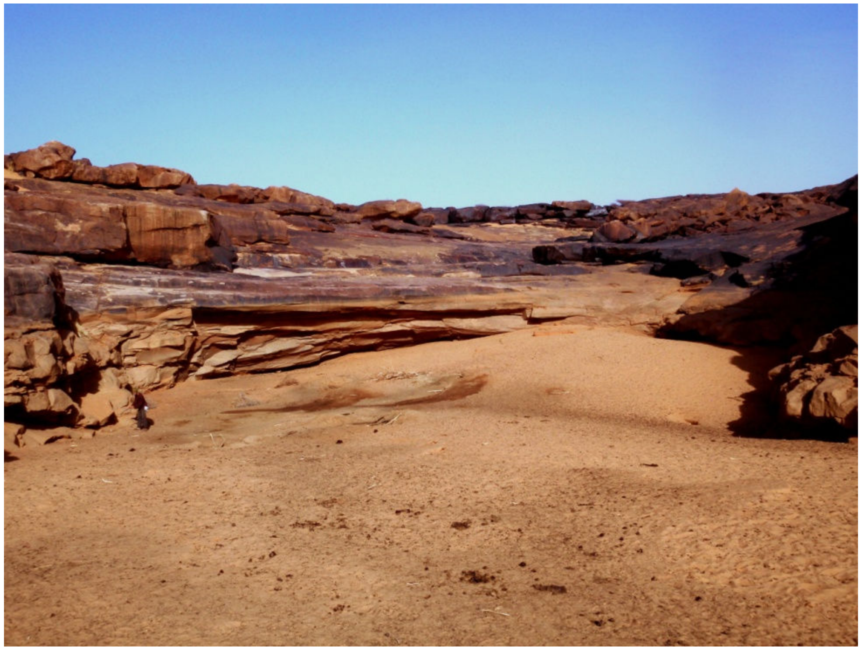

G66 Tin Waadine
Guelta was dry at time of visit
Photo: JC Brito

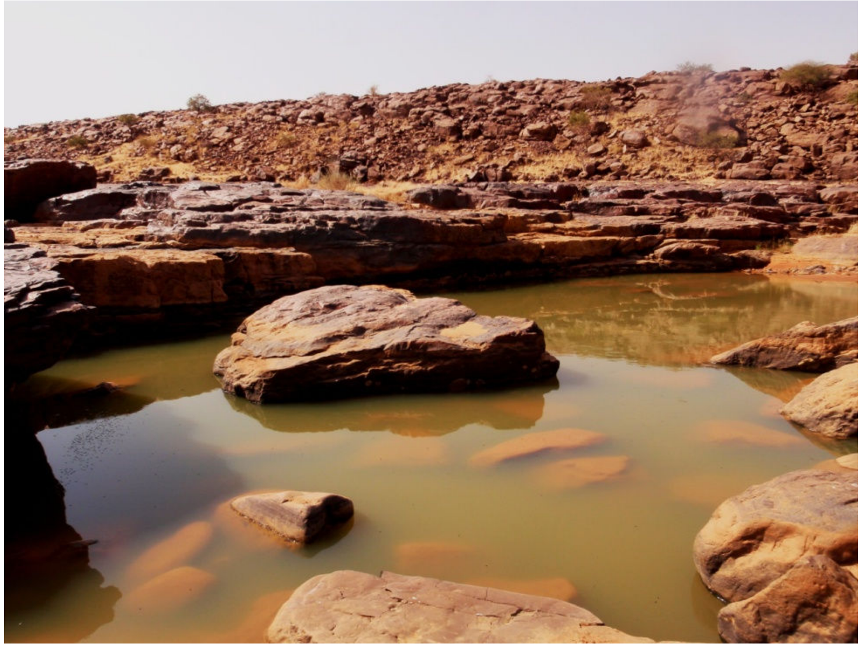

G67 Tkhsutin
Photo: JC Brito

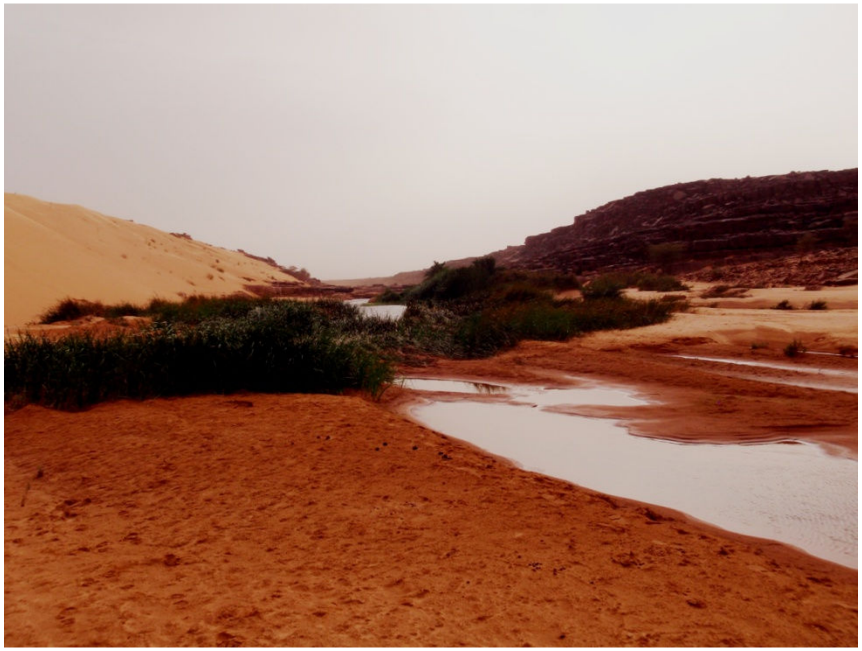

G68 Toumbahjît
Photo: JC Brito

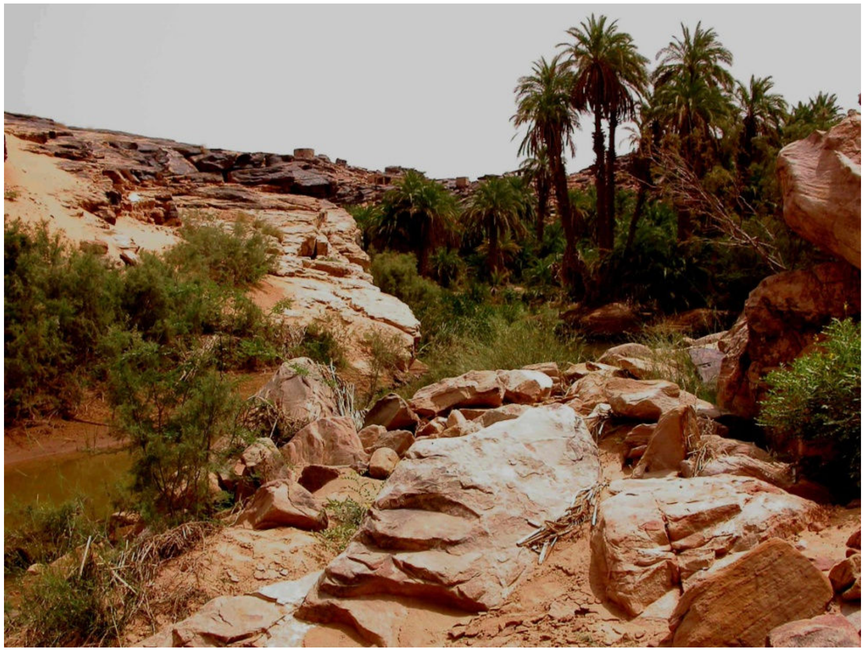

G69 Toûngâd
Guelta (not observable) is surrounded by dense vegetation
Photo: F Martínez-Freiría
