# Supplementary material for: Overlooked Mountain Rock Pools in Deserts Are Critical Local Hotspots of Biodiversity
Source: PLoS One. 2015 Feb 25;10(2):e0118367. doi: 10.1371/journal.pone.0118367 (PMC4340953; doi:10.1371/journal.pone.0118367)
Supplement: S1 Table — (PDF) [file pone.0118367.s002.pdf]

Table S1: Gueltas name, code and mountain. Seasonality character of each guelta.

Code	Name	Mountain	Seasonality
G01	Agmeimîne	Adrar Atar	Seasonal
G02	Ain El Berbera	Afollé	Seasonal
G03	Amzouzef	Tagant	Permanent
G04	Aouînet Nanâga	Assaba	Seasonal
G05	Aouînet Teidoûma	Tagant	Seasonal
G06	Aouînet Tenbouckit	Assaba	Seasonal
G07	Ayoûn en Na'aj	Afollé	Permanent
G08	Bâfa	Assaba	Seasonal
G09	Bajai	Tagant	Permanent
G10	Ch'Bayer	Tagant	Permanent
G11	Daal	Tagant	Seasonal
G12	Dâber	Tagant	Permanent
G13	Dâyet et Teila	Adrar Atar	Permanent
G14	Dekheïlet el ‘Aleïb (=Dekla, Ain Bâjed)	Tagant	Seasonal
G15	El Barda	Assaba	Permanent
G16	El Ghâira, source	Assaba	Permanent
G17	El Gleitât	Adrar Atar	Seasonal
G18	El Hnouk gorge	Adrar Atar	Seasonal
G19	El Housseînîya	Tagant	Permanent
G20	El Khedia	Tagant	Permanent
G21	El Mefga	Afollé	Permanent
G22	Emreimida	Tagant	Permanent
G23	E-n-Guinâr	Tagant	Seasonal
G24	Fanar	Tagant	Seasonal
G25	Foum el Kour	Tagant	Permanent
G26	Foum Goussas	Assaba	Permanent
G27	Galoûla	Assaba	Permanent
G28	Gamra Ouarbî	Tagant	Permanent
G29	Gânçai source	Assaba	Permanent
G30	Garaouel	Tagant	Permanent
G31	Glât el Bil	Adrar Atar	Seasonal
G32	Gleitat Ej Jmel	Tagant	Seasonal
G33	Goumbel	Assaba	Permanent
G34	Gueltet Thor	Assaba	Permanent
G35	Guenétir, source	Assaba	Permanent
G36	Guérou	Assaba	Seasonal
G37	Guidemballa	Assaba	Permanent
G38	Hamdoûn	Adrar Atar	Permanent
G39	Jabara	Tagant	Permanent
G40	Kabda	Tagant	Permanent
G41	Kaimel	Tagant	Seasonal
G42	Kediet El Grâne	Assaba	Seasonal
G43	Laout	Tagant	Permanent
G44	Laout, 1km S of	Tagant	Permanent
G45	Legleyta	Assaba	Seasonal
G46	Lemmollah	Tagant	Seasonal
G47	Leouel	Tagant	Seasonal
G48	Matmâta	Tagant	Permanent
G49	M'cherba	Tagant	Permanent
G50	Mendjoura	Tagant	Seasonal
G51	Metraoucha	Afollé	Permanent
G52	Meyla	Assaba	Permanent
G53	Oumm el Arjam	Tagant	Seasonal
G54	Oumm el Mhâr	Afollé	Permanent
G55	Oumm Icheglâne	Assaba	Permanent
G56	Oumm Icheglâne, 5km NW of	Assaba	Seasonal
G57	Oumm Lemhâr (=Molomhar)	Adrar Atar	Permanent
G58	Rh' Zembou	Tagant	Permanent
G59	Soufa, oued	Assaba	Permanent
G60	Suklan	Tagant	Seasonal
G61	Taorta	Tagant	Seasonal
G62	Tartêga	Tagant	Permanent
G63	Tartêga, upstream of	Tagant	Permanent
G64	Taoujafet	Tagant	Seasonal
G65	Terjît, oasis	Adrar Atar	Permanent
G66	Tin Waadine	Tagant	Seasonal
G67	Tkhsutin	Tagant	Permanent
G68	Toumbahjît	Adrar Atar	Permanent
G69	Toûngâd	Adrar Atar	Permanent
