# Supplementary material for: Overlooked Mountain Rock Pools in Deserts Are Critical Local Hotspots of Biodiversity
Source: PLoS One. 2015 Feb 25;10(2):e0118367. doi: 10.1371/journal.pone.0118367 (PMC4340953; doi:10.1371/journal.pone.0118367)
Supplement: S2 Table — (PDF) [file pone.0118367.s003.pdf]

Table S2: Taxa present in gueltas. Taxa scientific and common name, biogeographic affinity and IUCN status.

Group	Taxa	Common name	Affinity	IUCN	G01	G02	G03	G04	G05	G06	G07	G08	G09	G10	G11	G12	G13	G14	G15	G16	G17	G18	G19	G20	G21	G22	G23	G24	G25	G26	G27	G28	G29	G30	G31	G32	G33	G34	G35
Fishes	*Barbus sp.*	Barb	NA	NE	0	0	0	1	1	0	0	0	0	0	0	0	1	0	0	0	1	0	0	0	0	0	1	0	0	0	0	0	1	1	1	0	0	0	0
Fishes	*Brycinus nurse*	Nurse Tetra	Afro-tropical	NE	0	0	0	0	0	0	0	0	0	0	0	0	0	0	0	0	0	0	0	0	0	0	0	0	0	0	0	0	0	0	0	0	1	0	0
Fishes	*Clarias anguillaris*	Eel Catfish	Afro-tropical	NE	0	0	0	0	0	0	0	1	0	0	0	0	0	0	0	0	0	0	1	0	0	0	0	0	0	1	0	0	0	1	0	0	1	1	1
Fishes	*Sarotherodon spp.*	Tilapia	NA	NE	0	0	0	0	0	0	0	1	0	0	0	0	0	0	0	0	0	0	0	0	0	0	0	0	0	1	1	0	0	0	0	0	1	0	1
Fishes	*Schilbe sp.*	African butter catfish	Afro-tropical	NE	0	0	0	0	0	0	0	0	0	0	0	0	0	0	0	0	0	0	0	0	0	0	0	0	0	0	0	0	0	0	0	0	1	0	0
Amphibians	*Amietophrynus xeros*	Subdesert Toad	Sahelian endemic	LC	1	0	0	1	0	0	0	0	0	0	0	0	0	0	1	0	0	1	1	1	1	0	0	0	0	0	1	0	0	0	0	0	1	1	0
Amphibians	*Hoplobatrachus cf. occipitalis*	Crowned Frog	Mauritania endemic	NE	0	0	0	1	1	1	0	1	0	1	0	0	0	0	1	1	0	0	1	1	1	0	1	0	0	1	1	1	1	1	0	0	1	0	1
Amphibians	*Hoplobatrachus occipitalis*	African Groove-crowned Frog	Afro-tropical	LC	0	1	0	0	0	0	1	0	0	0	0	0	0	0	0	0	1	0	0	0	0	0	0	0	0	0	0	0	0	0	0	0	0	1	0
Amphibians	*Kassina senegalensis*	Senegal Land Frog	Afro-tropical	LC	0	0	0	0	0	0	0	0	0	0	0	0	0	0	0	0	0	0	0	0	0	0	0	0	0	0	0	0	0	0	0	0	0	0	0
Amphibians	*Phrynobatrachus spp.*	Puddle frog	Afro-tropical	NE	0	0	0	0	0	0	0	0	0	0	0	0	0	0	0	0	0	0	0	0	0	0	0	0	0	0	1	0	0	0	0	0	0	0	0
Amphibians	*Ptychadena spp.*	Grass frog	Afro-tropical	NE	0	0	0	0	0	0	0	0	0	0	0	0	0	0	0	0	0	0	0	0	0	0	0	0	0	0	0	0	0	0	0	0	0	0	1
Amphibians	*Tomopterna cryptotis*	Common Sand Frog	Afro-tropical	LC	0	0	0	0	0	0	0	0	0	0	0	0	0	0	0	0	0	0	0	0	0	0	0	0	0	0	0	0	0	0	0	0	0	0	0
Reptiles	*Acanthodactylus dumerili*	Duméril's Fringe-fingered Lizard	Sahara endemic	NE	0	0	0	0	0	0	0	0	0	0	0	0	1	0	0	0	0	0	0	0	0	0	0	0	0	0	0	0	0	0	0	0	0	0	0
Reptiles	*Agama boueti*	Bouet's Agama	Sahelian endemic	LC	0	0	0	0	0	0	0	0	0	0	0	0	0	0	0	0	0	0	0	0	0	0	0	0	0	0	0	0	0	1	0	0	0	0	0
Reptiles	*Agama boueti C*	Bouet's Agama	Mauritania endemic	LC	0	0	0	1	1	0	0	0	0	0	0	0	0	0	0	0	0	0	0	0	1	0	1	0	0	0	0	0	0	0	0	0	0	0	0
Reptiles	*Agama boueti W*	Bouet's Agama	Mauritania endemic	LC	0	0	0	0	0	0	0	0	0	0	0	0	0	0	0	0	0	0	0	0	0	0	0	0	0	0	0	0	0	0	0	0	0	1	0
Reptiles	*Agama boulengeri E*	Boulenger's Agama	Mauritania endemic	LC	0	1	0	0	0	0	1	0	0	0	0	0	0	0	0	0	0	0	0	0	1	0	0	0	0	0	0	0	0	0	0	0	0	0	0
Reptiles	*Agama boulengeri W*	Boulenger's Agama	Mauritania endemic	LC	0	0	1	1	1	1	0	0	1	1	1	1	1	1	1	1	1	0	1	1	0	1	1	1	1	1	1	0	1	1	1	1	1	1	1
Reptiles	*Atractaspis micropholis*	Sahelian Burrowing Asp	Sahelian endemic	LC	0	0	0	0	0	0	0	0	0	0	0	0	0	0	0	0	0	0	0	0	0	0	0	0	0	0	0	0	0	0	0	0	1	0	0
Reptiles	*Cerastes cerastes*	Saharan horned viper	Saharo-sindian	NE	0	0	1	0	0	0	0	0	0	0	0	0	0	0	0	0	0	0	0	0	0	0	0	0	0	0	0	0	0	0	0	0	0	0	0
Reptiles	*Chamaeleo africanus*	The Basilisk Chameleon	Afro-tropical	NE	0	0	0	0	0	0	0	0	0	0	0	0	0	0	0	0	0	0	0	0	1	0	0	0	0	0	0	0	0	0	0	0	0	0	0
Reptiles	*Crocodylus suchus*	West African crocodile	Afro-tropical	NE	0	1	1	1	1	0	0	1	1	1	0	0	0	0	0	1	0	0	1	1	1	1	1	0	0	1	1	0	0	1	0	1	1	1	1
Reptiles	*Echis leucogaster*	White-bellied Carpet Viper	Sahelian endemic	LC	0	0	0	0	0	0	0	0	0	0	0	0	0	0	1	0	0	0	0	0	0	0	0	0	0	0	0	0	0	1	0	0	0	0	0
Reptiles	*Bamanophis dorri*	Dorr's racer	Afro-tropical	NE	0	0	0	0	0	0	0	0	0	0	0	0	0	0	0	0	0	0	0	0	0	0	0	0	0	0	0	0	0	1	0	0	0	0	0
Reptiles	*Naja nigricollis*	Black-necked spitting cobra	Afro-tropical	NE	0	0	0	1	0	0	0	0	0	0	0	0	0	0	0	0	0	0	0	0	0	0	0	0	0	0	1	0	0	0	0	0	0	0	0
Reptiles	*Psammophis elegans*	Elegant Sand Racer	Afro-tropical	NE	0	0	0	1	0	0	0	0	0	0	0	0	0	0	1	0	0	0	0	0	0	0	0	0	0	0	0	0	0	0	0	0	1	0	0
Reptiles	*Ptyodactylus cf. togoensis*	Fan-fingered gecko	Mauritania endemic	NE	0	0	0	1	0	0	0	0	0	0	0	0	0	0	1	1	0	0	0	0	0	0	0	0	0	1	1	0	0	0	0	0	1	0	0
Reptiles	*Python sebae*	African rock python	Afro-tropical	NE	0	0	0	0	0	1	0	0	0	0	0	0	0	0	0	0	0	0	0	0	0	0	0	0	0	1	1	0	0	0	0	0	0	0	0
Reptiles	*Stenodactylus petrii*	Dune gecko	Sahara endemic	NE	0	0	0	0	0	0	0	0	0	0	0	0	0	0	0	0	0	0	0	0	0	0	0	0	0	0	0	0	0	0	0	1	0	0	0
Reptiles	*Tarentola hoggarensis*	African Wall Gecko	Afro-tropical	NE	0	0	0	1	0	0	1	0	1	0	0	0	0	0	0	0	0	0	0	0	0	0	0	0	0	0	0	0	0	0	0	1	0	0	0
Reptiles	*Tarentola parvicarinata*	Sierra Leone Wall Gecko	Sahelian endemic	NE	1	1	1	1	1	1	1	0	1	1	1	1	0	1	1	1	1	0	1	1	1	1	1	0	0	1	1	0	1	0	0	1	1	1	0
Reptiles	*Trachylepis quinquetaeniata*	African Five-lined Skink	Afro-tropical	NE	0	0	0	0	0	0	0	0	0	0	0	0	0	0	0	0	0	0	0	0	0	0	0	0	0	0	0	0	0	0	0	0	0	0	0
Reptiles	*Tropiocolotes tripolitanus*	Northern Sand Gecko	Sahara endemic	LC	1	0	0	1	0	1	1	0	0	0	0	0	0	0	1	1	0	0	0	0	0	0	0	0	0	0	0	0	0	0	0	0	0	0	0
Reptiles	*Uromastyx dispar*	Sudan Mastigure	Sahara endemic	NE	0	0	1	0	0	0	0	0	0	1	0	1	1	0	0	0	0	0	0	1	0	0	0	1	1	0	0	0	0	0	0	1	0	0	0
Reptiles	*Varanus exanthematicus*	Savannah Monitor	Afro-tropical	LC	0	0	0	0	0	0	0	0	0	0	0	0	0	0	1	0	0	0	0	0	0	0	0	0	0	0	0	0	0	0	0	0	0	0	0
Reptiles	*Varanus niloticus*	Nile monitor	Afro-tropical	NE	0	1	1	0	0	0	0	0	1	0	0	1	0	0	1	0	0	0	1	0	1	0	0	1	1	0	1	1	0	1	0	0	1	1	0
Mammals	*Acomys airensis*	Western Saharan Spiny Mouse	Sahelian endemic	LC	0	0	0	0	0	0	1	0	0	0	0	0	0	0	0	0	0	0	0	0	0	0	0	0	0	0	0	0	0	0	0	0	0	0	0
Mammals	*Asellia tridens*	Geoffroy's Trident Leaf-nosed Bat	Saharo-sindian	LC	0	0	0	0	0	0	0	0	0	0	0	0	0	0	0	0	0	0	0	0	0	0	0	0	0	0	0	0	0	0	0	0	0	0	1
Mammals	*Atelerix albiventris*	Four-toed Hedgehog	Afro-tropical	LC	0	0	0	0	0	0	0	0	0	0	0	0	0	0	0	0	0	0	0	0	0	0	0	0	0	0	0	0	0	0	0	0	0	0	0
Mammals	*Canis aureus*	Golden Jackal	Saharo-sindian	LC	0	0	1	0	1	0	0	1	1	1	0	0	0	0	1	0	0	0	0	0	1	0	1	0	0	0	1	0	0	1	0	1	1	0	0
Mammals	*Civettictis civetta*	African Civet	Afro-tropical	LC	0	0	0	0	0	0	0	0	0	0	0	0	0	0	0	0	0	0	0	0	0	0	0	0	0	0	0	0	0	0	0	0	0	0	0
Mammals	*Erythrocebus patas*	Erythrocebus patas	Afro-tropical	LC	0	0	0	0	0	0	1	1	0	0	0	0	0	0	0	0	0	0	0	0	0	0	0	0	0	0	0	0	0	0	0	0	0	0	1
Mammals	*Felis caracal*	Caracal	Afro-tropical	LC	0	0	0	0	0	0	0	0	0	0	0	0	0	0	1	0	0	0	0	0	0	0	0	0	0	0	0	0	0	0	0	0	0	0	0
Mammals	*Felovia vae*	Felou Gundi	Mauritania endemic	DD	0	0	0	1	0	1	1	0	1	0	1	1	0	1	1	0	0	0	1	0	1	0	0	0	0	1	1	1	1	1	0	1	1	0	1
Mammals	*Genetta genetta*	Common Genet	Afro-tropical	LC	0	0	0	0	0	0	0	0	0	0	0	0	0	0	0	0	0	0	0	0	0	0	0	0	0	1	0	0	0	0	0	0	0	1	0
Mammals	*Herpestes sanguinea*	Slender Mongoose	Afro-tropical	LC	0	0	0	0	0	0	0	0	0	0	0	0	0	0	1	0	0	0	0	0	0	0	0	0	0	0	0	0	0	0	0	0	0	0	0
Mammals	*Hystrix cristata*	Crested Porcupine	Afro-tropical	LC	0	0	0	0	1	0	1	0	0	0	0	0	0	1	0	0	0	0	1	0	1	0	1	0	0	0	0	0	0	0	0	0	1	0	0
Mammals	*Ichneumia albicauda*	White-tailed Mongoose	Afro-tropical	LC	0	0	0	0	0	0	0	0	0	0	0	0	0	0	0	0	0	0	0	0	0	0	0	0	0	0	0	0	0	0	0	0	0	0	0
Mammals	*Lepus sp.*	African hare	Palaearctic	LC	0	0	0	0	0	0	0	0	0	0	0	0	0	0	0	0	0	0	0	0	0	0	0	0	0	0	0	0	0	0	0	0	0	0	0
Mammals	*Mellivora capensis*	Honey Badger	Afro-tropical	LC	0	0	0	0	0	0	0	0	0	0	0	0	0	0	0	0	0	0	0	0	0	0	0	0	0	0	0	0	0	0	0	0	0	0	0
Mammals	*Papio papio*	Guinea baboon	Afro-tropical	NT	0	1	0	1	1	0	1	1	1	0	0	0	0	0	1	0	0	0	0	0	0	0	1	1	1	1	1	0	1	1	0	0	1	0	1
Mammals	*Paraechinus aethiopicus*	Desert Hedgehog	Saharo-sindian	LC	0	0	0	0	0	0	1	0	0	0	0	0	0	0	0	0	0	0	0	0	0	0	0	0	0	0	0	0	0	0	0	0	0	0	0
Mammals	*Phacochoerus africanus*	Common Warthog	Afro-tropical	LC	0	0	0	0	0	0	0	0	0	0	0	0	0	0	0	0	0	0	0	0	0	0	0	0	0	0	0	0	0	1	0	0	0	0	0
Mammals	*Praomys sp.*	Long-footedd rat	Afro-tropical	LC	0	0	0	0	0	0	1	0	0	0	0	0	0	0	0	0	0	0	0	0	0	0	0	0	0	0	0	0	0	0	0	0	0	0	0
Mammals	*Procavia capensis*	Rock Hyrax	Afro-tropical	LC	0	1	0	1	0	0	1	0	1	0	1	1	0	0	0	0	0	0	1	1	1	0	0	0	0	1	0	1	0	1	0	1	1	0	0
Mammals	*Rhinopoma hardwickei*	Lesser Mouse-tailed Bat	Saharo-sindian	LC	0	0	0	0	0	0	0	0	1	0	0	0	0	0	0	0	0	0	0	0	0	0	0	0	0	0	0	0	0	0	0	0	0	0	0
Mammals	*Vulpes pallida*	Pale Fox	Sahelian endemic	DD	0	0	0	1	0	0	0	0	0	0	0	0	0	0	0	0	0	0	0	0	0	0	0	1	0	0	0	0	0	0	0	0	0	0	0
Mammals	*Vulpes rueppellii*	Rüppel's Fox	Saharo-sindian	LC	0	0	0	0	0	0	0	0	0	0	0	0	0	0	0	0	0	0	0	0	0	0	0	0	0	0	0	0	0	0	0	0	0	1	0
Mammals	*Xerus erythropus*	Striped Ground Squirrel	Afro-tropical	LC	0	0	0	0	0	0	1	0	0	0	0	0	0	1	0	0	0	0	1	0	0	0	0	1	0	0	0	0	0	0	0	1	1	1	1

Group	Taxa	Common name	Affinity	IUCN	G36	G37	G38	G39	G40	G41	G42	G43	G44	G45	G46	G47	G48	G49	G50	G51	G52	G53	G54	G55	G56	G57	G58	G59	G60	G61	G62	G63	G64	G65	G66	G67	G68	G69
Fishes	*Barbus sp.*	Barb	NA	NE	0	0	1	0	0	0	0	0	0	0	0	1	0	0	1	0	0	0	1	0	0	1	0	0	0	0	0	0	0	1	0	0	0	1
Fishes	*Brycinus nurse*	Nurse Tetra	Afro-tropical	NE	0	0	0	0	0	0	0	0	0	0	0	0	0	0	0	0	0	0	0	0	0	0	0	0	0	0	0	0	0	0	0	0	0	0
Fishes	*Clarias anguillaris*	Eel Catfish	Afro-tropical	NE	0	1	0	1	0	0	0	0	0	0	0	0	1	0	0	1	0	0	0	0	0	1	0	1	0	0	1	0	0	0	0	0	0	0
Fishes	*Sarotherodon spp.*	Tilapia	NA	NE	0	1	0	0	0	0	0	0	0	0	0	0	0	0	0	1	0	0	0	0	0	0	0	0	0	0	0	0	0	0	0	0	0	0
Fishes	*Schilbe sp.*	African butter catfish	Afro-tropical	NE	0	0	0	0	0	0	0	0	0	0	0	0	0	0	0	0	0	0	0	0	0	0	0	0	0	0	0	0	0	0	0	0	0	0
Amphibians	*Amietophrynus xeros*	Subdesert Toad	Sahelian endemic	LC	0	0	0	1	0	0	0	1	1	0	0	0	1	0	0	1	1	1	1	1	0	1	0	1	0	0	1	0	1	1	0	0	1	1
Amphibians	*Hoplobatrachus cf. occipitalis*	Crowned Frog	Mauritania endemic	NE	1	1	0	1	0	0	0	1	1	1	0	1	1	0	1	1	1	0	1	1	1	0	0	1	0	0	1	1	0	0	1	1	0	0
Amphibians	*Hoplobatrachus occipitalis*	African Groove-crowned Frog	Afro-tropical	LC	0	0	1	0	0	0	0	0	0	0	0	0	0	0	0	0	0	0	0	0	0	1	0	0	0	0	0	0	0	1	0	0	1	0
Amphibians	*Kassina senegalensis*	Senegal Land Frog	Afro-tropical	LC	0	0	0	0	0	0	0	0	0	0	0	0	0	0	0	0	0	0	0	0	0	0	0	1	0	0	0	0	0	0	0	0	0	0
Amphibians	*Phrynobatrachus spp.*	Puddle frog	Afro-tropical	NE	0	0	0	0	0	0	0	0	0	0	0	0	0	0	0	0	1	0	0	0	0	0	0	0	0	0	0	0	0	0	0	0	0	0
Amphibians	*Ptychadena spp.*	Grass frog	Afro-tropical	NE	0	0	0	0	0	0	0	0	0	0	0	0	0	0	0	0	1	0	0	0	0	0	0	0	0	0	0	0	0	0	0	0	0	0
Amphibians	*Tomopterna cryptotis*	Common Sand Frog	Afro-tropical	LC	0	0	0	0	0	0	0	0	0	0	0	0	0	0	0	0	0	0	0	0	0	0	0	0	0	0	0	0	1	0	0	0	0	0
Reptiles	*Acanthodactylus dumerili*	Duméril's Fringe-fingered Lizard	Sahara endemic	NE	0	0	0	0	0	0	0	0	0	0	0	0	0	0	1	0	0	0	0	0	0	0	0	0	0	0	0	0	0	0	0	0	0	0
Reptiles	*Agama boueti*	Bouet's Agama	Sahelian endemic	LC	0	0	0	0	0	0	0	0	0	0	0	0	0	0	0	0	0	0	0	0	0	0	0	0	0	0	0	0	0	0	0	0	0	0
Reptiles	*Agama boueti C*	Bouet's Agama	Mauritania endemic	LC	0	0	0	0	0	0	0	0	0	0	0	0	0	0	0	0	0	0	0	0	0	0	0	0	0	0	0	0	0	0	0	0	0	0
Reptiles	*Agama boueti W*	Bouet's Agama	Mauritania endemic	LC	0	0	0	0	0	0	0	0	0	0	0	0	0	0	0	0	0	0	0	0	0	0	0	0	0	0	0	0	0	0	0	0	0	0
Reptiles	*Agama boulengeri E*	Boulenger's Agama	Mauritania endemic	LC	0	0	0	0	0	0	0	0	0	0	0	0	0	0	0	1	0	0	1	0	0	0	0	0	0	0	0	0	0	0	0	0	0	0
Reptiles	*Agama boulengeri W*	Boulenger's Agama	Mauritania endemic	LC	1	1	0	1	1	1	1	1	1	0	1	1	1	1	1	0	1	1	0	1	1	1	1	1	1	1	1	1	1	1	1	1	0	1
Reptiles	*Atractaspis micropholis*	Sahelian Burrowing Asp	Sahelian endemic	LC	0	0	0	0	0	0	0	0	0	0	0	0	0	0	0	0	0	0	0	0	0	0	0	1	0	0	0	0	0	0	0	0	0	0
Reptiles	*Cerastes cerastes*	Saharan horned viper	Saharo-sindian	NE	0	0	0	0	0	0	0	0	0	0	0	0	0	0	0	0	0	0	0	0	0	0	0	0	0	0	0	0	0	0	0	0	0	0
Reptiles	*Chamaeleo africanus*	The Basilisk Chameleon	Afro-tropical	NE	0	0	0	0	0	0	0	0	0	0	0	0	0	0	0	0	0	0	0	0	0	0	0	0	0	0	0	0	0	0	0	0	0	0
Reptiles	*Crocodylus suchus*	West African crocodile	Afro-tropical	NE	0	1	0	1	1	0	0	0	0	1	0	0	1	1	0	1	1	0	1	1	1	0	1	1	1	1	1	1	0	0	1	1	0	0
Reptiles	*Echis leucogaster*	White-bellied Carpet Viper	Sahelian endemic	LC	0	0	0	0	0	0	0	1	1	0	0	0	0	0	0	0	1	0	0	0	0	0	0	0	0	1	0	0	0	0	1	0	0	0
Reptiles	*Bamanophis dorri*	Dorr's racer	Afro-tropical	NE	0	0	0	0	0	0	0	0	0	0	0	0	0	0	0	0	1	0	1	0	0	0	0	0	0	0	0	0	0	0	0	0	0	0
Reptiles	*Naja nigricollis*	Black-necked spitting cobra	Afro-tropical	NE	0	0	0	0	0	0	0	0	0	0	0	0	0	0	0	0	1	0	0	0	0	0	0	0	0	0	0	0	0	0	0	0	0	0
Reptiles	*Psammophis elegans*	Elegant Sand Racer	Afro-tropical	NE	0	0	0	0	0	0	0	0	0	0	0	0	0	0	0	0	0	0	0	1	1	0	0	1	0	0	0	0	0	0	0	0	0	0
Reptiles	*Ptyodactylus cf. togoensis*	Fan-fingered gecko	Mauritania endemic	NE	0	0	0	1	0	0	0	1	1	0	0	0	1	0	0	0	1	0	0	1	1	0	0	1	0	0	1	1	0	0	1	0	0	0
Reptiles	*Python sebae*	African rock python	Afro-tropical	NE	0	0	0	0	0	0	0	0	0	0	0	0	0	0	0	1	1	0	1	1	1	0	0	0	0	0	0	0	0	0	0	0	0	0
Reptiles	*Stenodactylus petrii*	Dune gecko	Sahara endemic	NE	0	0	0	0	0	0	0	0	0	0	0	0	0	0	0	0	0	0	0	0	0	0	0	0	0	0	0	0	0	0	0	0	0	0
Reptiles	*Tarentola hoggarensis*	African Wall Gecko	Afro-tropical	NE	0	0	0	0	0	1	0	0	0	0	0	0	0	1	0	0	0	0	0	0	0	0	0	0	0	1	1	1	0	0	1	0	0	0
Reptiles	*Tarentola parvicarinata*	Sierra Leone Wall Gecko	Sahelian endemic	NE	1	1	0	1	1	1	1	1	1	1	1	0	1	1	1	1	1	1	1	1	1	0	1	1	1	1	1	1	0	1	1	1	0	0
Reptiles	*Trachylepis quinquetaeniata*	African Five-lined Skink	Afro-tropical	NE	0	0	0	0	0	0	0	0	0	0	0	0	0	0	0	0	1	0	0	0	0	0	0	0	0	0	0	0	0	0	0	0	0	0
Reptiles	*Tropiocolotes tripolitanus*	Northern Sand Gecko	Sahara endemic	LC	0	1	0	0	0	0	0	0	0	0	1	0	0	0	0	0	0	0	0	0	0	0	0	1	0	1	0	0	0	0	0	0	0	0
Reptiles	*Uromastyx dispar*	Sudan Mastigure	Sahara endemic	NE	0	0	0	0	0	0	0	0	0	0	0	0	0	0	0	0	0	1	0	0	0	0	1	0	1	1	0	0	0	0	1	1	0	0
Reptiles	*Varanus exanthematicus*	Savannah Monitor	Afro-tropical	LC	0	0	0	0	0	0	0	0	0	0	0	0	0	0	0	0	0	0	0	0	0	0	0	0	0	0	0	0	0	0	1	0	0	0
Reptiles	*Varanus niloticus*	Nile monitor	Afro-tropical	NE	0	0	0	1	0	0	0	1	1	1	1	0	1	1	1	1	1	0	1	0	0	0	0	1	1	0	1	1	0	0	1	1	0	0
Mammals	*Acomys airensis*	Western Saharan Spiny Mouse	Sahelian endemic	LC	0	0	0	0	0	0	0	0	0	0	0	0	0	0	0	0	0	0	0	0	0	0	0	0	0	0	0	0	0	0	0	0	0	0
Mammals	*Asellia tridens*	Geoffroy's Trident Leaf-nosed Bat	Saharo-sindian	LC	0	0	0	0	0	0	0	0	0	0	0	0	0	0	0	0	0	0	0	0	0	0	0	0	0	0	0	0	0	0	0	0	0	0
Mammals	*Atelerix albiventris*	Four-toed Hedgehog	Afro-tropical	LC	0	0	0	0	0	0	0	0	0	0	0	0	0	0	0	0	0	0	0	0	0	0	0	0	0	1	0	0	0	0	0	0	0	0
Mammals	*Canis aureus*	Golden Jackal	Saharo-sindian	LC	0	0	0	1	1	0	0	1	1	1	0	0	1	1	0	1	0	0	0	0	0	1	1	1	0	0	1	1	0	0	1	0	0	0
Mammals	*Civettictis civetta*	African Civet	Afro-tropical	LC	0	0	0	0	0	0	0	0	0	0	0	0	0	0	0	0	0	0	1	0	0	0	0	0	0	0	0	0	0	0	0	0	0	0
Mammals	*Erythrocebus patas*	Erythrocebus patas	Afro-tropical	LC	0	0	0	1	0	0	0	0	0	0	0	0	1	0	0	0	1	0	0	0	0	0	0	0	0	0	1	1	0	0	0	0	0	0
Mammals	*Felis caracal*	Caracal	Afro-tropical	LC	0	0	0	0	0	0	0	0	0	0	0	0	0	0	0	0	0	0	0	0	0	0	0	0	0	0	0	0	0	0	0	0	0	0
Mammals	*Felovia vae*	Felou Gundi	Mauritania endemic	DD	0	1	1	1	0	0	1	1	1	0	1	0	1	1	0	0	1	0	1	0	1	1	0	1	1	1	1	1	1	1	1	1	0	1
Mammals	*Genetta genetta*	Common Genet	Afro-tropical	LC	0	0	0	0	0	0	0	0	0	0	0	0	0	0	0	0	0	0	0	0	0	0	0	0	0	0	0	0	0	0	0	0	0	0
Mammals	*Herpestes sanguinea*	Slender Mongoose	Afro-tropical	LC	0	0	0	0	0	0	1	0	0	0	0	0	0	0	0	0	0	0	0	0	0	0	0	0	0	0	0	0	0	0	0	0	0	0
Mammals	*Hystrix cristata*	Crested Porcupine	Afro-tropical	LC	0	0	0	0	0	0	0	0	0	0	0	1	0	0	0	0	1	0	0	0	0	0	0	1	1	1	0	0	0	0	1	1	0	0
Mammals	*Ichneumia albicauda*	White-tailed Mongoose	Afro-tropical	LC	0	0	0	0	0	0	0	0	0	0	0	0	0	0	0	0	0	0	1	0	0	0	0	0	0	0	0	0	0	0	0	0	0	0
Mammals	*Lepus sp.*	African hare	Palaearctic	LC	0	0	0	0	0	0	0	0	0	0	0	0	0	0	1	0	0	0	0	0	0	0	0	0	0	0	0	0	0	0	0	0	0	0
Mammals	*Mellivora capensis*	Honey Badger	Afro-tropical	LC	0	0	0	0	0	0	0	0	0	1	0	0	0	0	0	0	0	0	0	0	0	0	0	0	0	0	0	0	0	0	0	0	0	0
Mammals	*Papio papio*	Guinea baboon	Afro-tropical	NT	1	0	0	1	0	0	1	1	1	1	0	0	1	1	0	1	1	0	1	1	1	0	0	1	0	0	1	1	0	0	0	0	0	0
Mammals	*Paraechinus aethiopicus*	Desert Hedgehog	Saharo-sindian	LC	0	0	0	0	0	0	0	0	0	0	0	0	0	0	0	0	0	0	0	0	0	0	0	0	0	0	0	0	1	0	0	0	0	0
Mammals	*Phacochoerus africanus*	Common Warthog	Afro-tropical	LC	0	0	0	0	0	0	0	0	0	0	0	0	0	0	0	0	0	0	0	0	0	0	0	0	0	0	0	0	0	0	0	0	0	0
Mammals	*Praomys sp.*	Long-footedd rat	Afro-tropical	LC	0	0	0	0	0	0	0	0	0	0	0	0	0	0	0	0	0	0	0	0	0	0	0	0	0	0	0	0	0	0	0	0	0	0
Mammals	*Procavia capensis*	Rock Hyrax	Afro-tropical	LC	1	0	1	1	0	0	0	1	1	0	0	1	1	1	0	1	1	0	1	0	0	0	0	1	0	0	1	1	0	1	0	0	0	0
Mammals	*Rhinopoma hardwickei*	Lesser Mouse-tailed Bat	Saharo-sindian	LC	0	0	0	1	0	0	0	0	0	0	0	0	1	1	0	0	0	0	0	0	0	0	0	0	0	0	1	1	0	0	0	0	0	0
Mammals	*Vulpes pallida*	Pale Fox	Sahelian endemic	DD	0	0	0	0	0	0	0	0	0	0	0	0	0	0	0	1	0	0	0	0	0	0	0	0	0	0	0	0	0	0	0	0	0	0
Mammals	*Vulpes rueppellii*	Rüppel's Fox	Saharo-sindian	LC	0	0	0	0	0	0	0	0	0	0	0	0	0	0	0	0	0	0	0	0	0	0	0	0	0	0	0	0	0	0	0	0	0	0
Mammals	*Xerus erythropus*	Striped Ground Squirrel	Afro-tropical	LC	0	0	0	0	0	0	0	0	0	0	0	0	0	0	1	0	0	0	0	0	0	0	0	1	1	0	0	0	0	0	0	1	0	0
