# Supplementary material for: Overlooked Mountain Rock Pools in Deserts Are Critical Local Hotspots of Biodiversity
Source: PLoS One. 2015 Feb 25;10(2):e0118367. doi: 10.1371/journal.pone.0118367 (PMC4340953; doi:10.1371/journal.pone.0118367)
Supplement: S3 Table — (PDF) [file pone.0118367.s004.pdf]

Table S3: Number of species and endemics present in each guelta by taxonomic group and IUCN status. Sum of species present in gueltas of each mountain. Gueltas code. DD: Data deficient; NE: Not evaluated; LC: Least concern; NT: Near threatened; VU: Vulnerable; CR: Critically Endangered.

Code	Fishes	Amphibians	Reptiles	Mammals	Total	Mauritania Endemics	DD	NE	LC	NT	VU	CR
G01	0	1	2	0	3	0	0	1	2	0	0	0
G13	1	0	3	0	4	1	0	3	1	0	0	0
G17	1	1	2	0	4	1	0	2	2	0	0	0
G18	0	1	0	0	1	0	0	0	1	0	0	0
G31	1	0	1	0	2	1	0	1	1	0	0	0
G38	1	1	0	2	4	1	1	1	2	0	0	0
G57	2	2	1	2	7	2	1	2	4	0	0	0
G65	1	2	2	2	7	2	1	2	4	0	0	0
G68	0	2	0	0	2	0	0	0	2	0	0	0
G69	1	1	1	1	4	2	1	1	2	0	0	0
Σ Adrar	2	2	5	3	12	2	1	5	6	0	0	0
G03	0	0	6	1	7	1	0	5	2	0	0	0
G05	1	1	4	3	9	3	0	4	4	1	0	0
G09	0	0	5	5	10	2	1	4	4	1	0	0
G10	0	1	4	1	6	2	0	4	2	0	0	0
G11	0	0	2	2	4	2	1	1	2	0	0	0
G12	0	0	4	2	6	2	1	3	2	0	0	0
G14	0	0	2	3	5	2	1	1	3	0	0	0
G19	1	2	4	4	11	3	1	5	5	0	0	0
G20	0	2	4	1	7	2	0	4	3	0	0	0
G22	0	0	3	0	3	1	0	2	1	0	0	0
G23	1	1	4	3	9	3	0	4	4	1	0	0
G24	0	0	3	3	6	1	1	2	2	1	0	0
G25	0	0	3	1	4	1	0	2	1	1	0	0
G28	0	1	1	2	4	2	1	2	1	0	0	0
G30	2	1	6	5	14	3	1	6	6	1	0	0
G32	0	0	6	4	10	2	1	5	4	0	0	0
G39	1	2	5	6	14	4	1	6	6	1	0	0
G40	0	0	3	1	4	1	0	2	2	0	0	0
G41	0	0	3	0	3	1	0	2	1	0	0	0
G43	0	2	5	4	11	4	1	4	5	1	0	0
G44	0	2	5	4	11	4	1	4	5	1	0	0
G46	0	0	4	1	5	2	1	2	2	0	0	0
G47	1	1	1	2	5	2	0	2	3	0	0	0
G48	1	2	5	6	14	4	1	6	6	1	0	0
G49	0	0	5	5	10	2	1	4	4	1	0	0
G50	1	1	4	2	8	2	0	5	3	0	0	0
G53	0	1	3	0	4	1	0	2	2	0	0	0
G58	0	0	4	1	5	1	0	3	2	0	0	0
G60	0	0	5	3	8	2	1	4	3	0	0	0
G61	0	0	7	3	10	2	1	4	5	0	0	0
G62	1	2	6	6	15	4	1	7	6	1	0	0
G63	0	1	6	6	13	4	1	6	5	1	0	0
G64	0	2	1	2	5	2	1	0	4	0	0	0
G66	0	1	9	3	13	4	1	7	5	0	0	0
G67	0	1	5	3	9	3	1	5	3	0	0	0
Σ Tagant	2	3	16	13	34	5	2	13	18	1	0	0
G04	1	2	9	4	16	5	2	8	5	1	0	0
G06	0	1	4	1	6	3	1	3	2	0	0	0
G08	2	1	1	3	7	1	0	4	2	1	0	0
G15	0	2	8	5	15	4	1	5	8	1	0	0
G16	0	1	5	0	6	3	0	4	2	0	0	0
G26	2	1	5	4	12	4	1	7	3	1	0	0
G27	1	3	7	3	14	4	1	9	3	1	0	0
G29	1	1	2	2	6	3	1	3	1	1	0	0
G33	4	2	7	6	19	4	1	10	7	1	0	0
G34	1	2	5	3	11	2	0	4	7	0	0	0
G35	2	2	2	5	11	3	1	5	4	1	0	0
G36	0	1	2	2	5	2	0	2	2	1	0	0
G37	2	1	4	1	8	3	1	5	2	0	0	0
G42	0	0	2	3	5	2	1	1	2	1	0	0
G45	0	1	3	3	7	1	0	4	2	1	0	0
G52	0	4	10	5	19	4	1	11	6	1	0	0
G55	0	2	6	1	9	3	0	6	2	1	0	0
G56	0	1	6	2	9	4	1	6	1	1	0	0
G59	1	3	8	6	18	4	1	7	9	1	0	0
Σ Assaba	5	6	17	14	42	6	2	18	21	1	0	0
G02	0	1	4	2	7	1	0	3	3	1	0	0
G07	0	1	4	9	14	2	1	2	10	1	0	0
G21	0	2	6	4	12	4	1	5	6	0	0	0
G51	2	2	5	4	13	2	1	7	4	1	0	0
G54	1	2	6	5	14	3	1	7	5	1	0	0
Σ Afollé	3	3	10	13	29	4	2	11	15	1	0	0
